# Fitness consequences of artificial selection on relative male genital size

**DOI:** 10.1038/ncomms11597

**Published:** 2016-05-18

**Authors:** Isobel Booksmythe, Megan L. Head, J. Scott Keogh, Michael D. Jennions

**Affiliations:** 1 Evolution, Ecology and Genetics, Research School of Biology, The Australian National University, Building 116, Daley Road, Acton, Canberra, Australian Capital Territory 2601, Australia; 2 Centre of Excellence in Biological Interactions Research, Department of Evolutionary Biology and Environmental Studies, University of Zürich, Winterthurerstrasse 190, Zürich CH-8057, Switzerland

## Abstract

Male genitalia often show remarkable differences among related species in size, shape and complexity. Across poeciliid fishes, the elongated fin (gonopodium) that males use to inseminate females ranges from 18 to 53% of body length. Relative genital size therefore varies greatly among species. In contrast, there is often tight within-species allometric scaling, which suggests strong selection against genital–body size combinations that deviate from a species' natural line of allometry. We tested this constraint by artificially selecting on the allometric intercept, creating lines of males with relatively longer or shorter gonopodia than occur naturally for a given body size in mosquitofish, *Gambusia holbrooki*. We show that relative genital length is heritable and diverged 7.6–8.9% between our up-selected and down-selected lines, with correlated changes in body shape. However, deviation from the natural line of allometry does not affect male success in assays of attractiveness, swimming performance and, crucially, reproductive success (paternity).

## Abstract (web summary)

Within species, there tends to be a tight relationship between genital size and body size, suggesting strong stabilizing selection. Here, Booksmythe *et al.* artificially select relative genital size in mosquitofish and find that novel genital size-body size combinations do not lead to expected fitness reductions.

## Graphical abstract

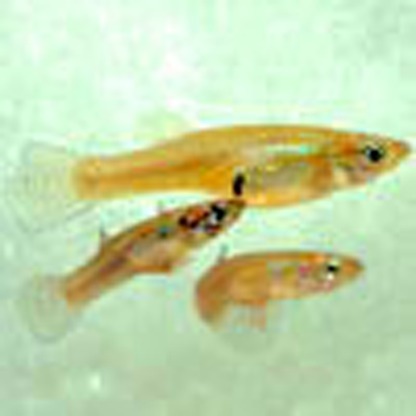

Male genitalia are remarkable for their extreme divergence among species in size, shape and complexity^1^,^2^,^3^. Despite high variation among species in mean relative genital size, within-species body-size scaling (static allometry) is very tight in some species; most of the variation in genital size is explained by variation in body size (that is, there is a high *R*^2^ value for the regression of genital on body size). If there is genetic variation for relative trait size, because males vary in their ontogenetic allometric slopes and/or intercepts, then without strong selection on deviations from the natural allometric relationship genetic drift should reduce *R*^2^. Tight allometry implies that males with novel trait–body size combinations (relatively large or small genitals for their body size) have lower fitness.

In live-bearing poeciliid fishes, males use their modified anal fin (gonopodium) to inseminate females. Gonopodium length varies across species from 18 to 53% of mean body size^4^. However, as in other taxa, there is often low intraspecific variation in genital length for a given body size^5^. For example, in the mosquitofish *G. holbrooki*, body length explains over 90% of variation in gonopodium length. Even so, recent selection analyses of poeciliids find that relative genital size and shape is associated with male mating and/or reproductive success in both *G. holbrooki*^6^,^7^ and a related species, the guppy, *Poecilia reticulata*^8^,^9^. Surprisingly, the detected selection is directional rather than stabilizing and favours males with a relatively large gonopodium for their body size.

A weakness of selection analyses is that they are correlational. A relationship between a focal trait and reproductive success can arise if both are affected by another variable^10^. In red deer, *Cervus elaphus*, for example, favourable environmental conditions lead to larger male antlers, but also elevate female breeding success, generating a spuriously high estimate of the selection gradient on male antler size^10^. How then do we determine whether relative genital size in poeciliids is under selection, which might account for its precise relationship with body size? One approach, especially in sexual selection studies, is to experimentally manipulate a focal trait^11^,^12^. To date, the only experimental evidence for direct selection on male genitals comes from gross manipulation of genital features by ablating or surgically removing major components. These studies demonstrate that certain genital traits affect fertilization success^13^,^14^,^15^,^16^,^17^,^18^,^19^. The problem with such experiments is that developmentally integrated traits that might affect the consequences of manipulating a single trait are left unchanged^2^,^20^,^21^. A reduction in male fitness might therefore reflect a lack of compensatory developmental changes in other traits rather than sexual selection acting directly on the manipulated trait^2^,^20^.

Crucially, we lack experimental studies in which novel genital–body size combinations are created such that developmentally integrated correlated traits can still co-evolve. An alternative approach to achieve this is to use artificial selection and compare the fitness of control and selected lineages. If a focal trait is heritable, artificial selection might even shift the mean value outside the natural range but, importantly, genetically or developmentally correlated traits will change in concert. Any resultant effects on fitness are therefore not attributed to a mismatch in the expression of co-evolved traits. Artificial selection should reduce fitness if the focal trait is under strong natural and/or sexual selection^21^.

Artificial selection on allometric intercepts or slopes to alter trait–body size relationships has been applied to naturally selected traits^21^,^22^,^23^, but surprisingly few studies have done so for putative sexually selected traits. The available studies have targeted traits such as ornaments, testes and weaponry that are assumed to affect male mating rate, sperm competitiveness and fighting success, respectively^24^,^25^,^26^,^27^,^28^,^29^. Alternatively, researchers have selected on net male attractiveness^30^,^31^. Only one study has selected for an aspect of male genitalia, namely absolute, but not body size-corrected, genital spine size in a beetle^13^. Surprisingly, given the ubiquity of high interspecific variation in genital size in many taxa, there are no studies using artificial selection to create males with novel combinations of genital and body size (outside the natural range of variation for the species in question, although these combinations might occur in closely related species). Creating these novel phenotypes is most readily achieved by selecting on the allometric intercept^32^,^33^. In principle, it could also be achieved by only selecting on the allometric slope such that mean relative trait size stays unchanged (so relative trait size increases for some males and decreases for others, depending on whether they are of larger or smaller than average body size). Only three studies have selected on allometric slopes in this way^34^,^35^,^36^. Theory suggests that it is more difficult to change allometric slopes than intercepts^22^,^23^.

To test whether there is strong sexual and/or natural selection on relative genital size, we artificially selected on the intercept of the allometric regression to either increase or decrease mean relative gonopodium length in *G. holbrooki* (three replicates of up-selected, down-selected and control lines). The tight relationship between gonopodium length and body size raises questions about the role of current selection against deviations from the natural line of allometry versus past selection for developmental trajectories that generates strong covariance between traits. After we applied artificial selection for eight generations, gonopodium length had diverged by 7.6–8.9% between our up-selected and down-selected lines. Mean relative gonopodium length changed in the direction of selection, while the allometric slope remained unchanged. We then tested whether deviations in either direction away from the natural line of allometry affect male fitness. Previous studies have reported directional selection on relative gonopodium length, but we did not find that males from up-selected lines are more attractive to females^11^,^12^ nor that they have weaker swimming performance^12^ than down-selected males. In combination, we did not find that the net effect of selection is greater male reproductive success (fitness) for control line males than males in either the up-selected or down-selected lines when they freely compete for mates and fertilization opportunities in semi-natural pools. In short, we did not find that novel genital–body size combinations are selected against.

## Results

### Evolution of relative genital size

**Figure 1:**
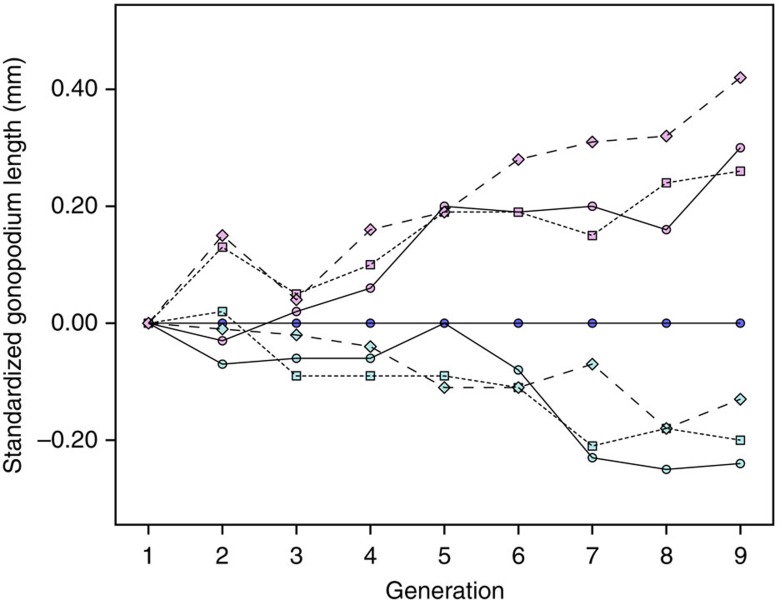
Mean gonopodium length for an average-sized male. Least square mean values were obtained from a GLM of all males from generations 1–9 (Log gonopodium length=Log SL+LineID (where LineID is each unique generation-replicate treated as a factor, *N*=75 levels). Selected line values are then plotted as deviations (mm) from the control mean for the relevant replicate and generation. Purple, Up; dark blue, Control; light blue, Down. Replicate *A*: circles and solid line, Replicate *B*: squares and dotted line; Replicate *C*: diamonds and dashed line. The line mean estimates are very precise due to the high *R*^2^ values and large sample sizes of each generation; thus, for clarity, s.e. bars are not presented (all s.e. are<0.02 mm).

**Figure 2:**
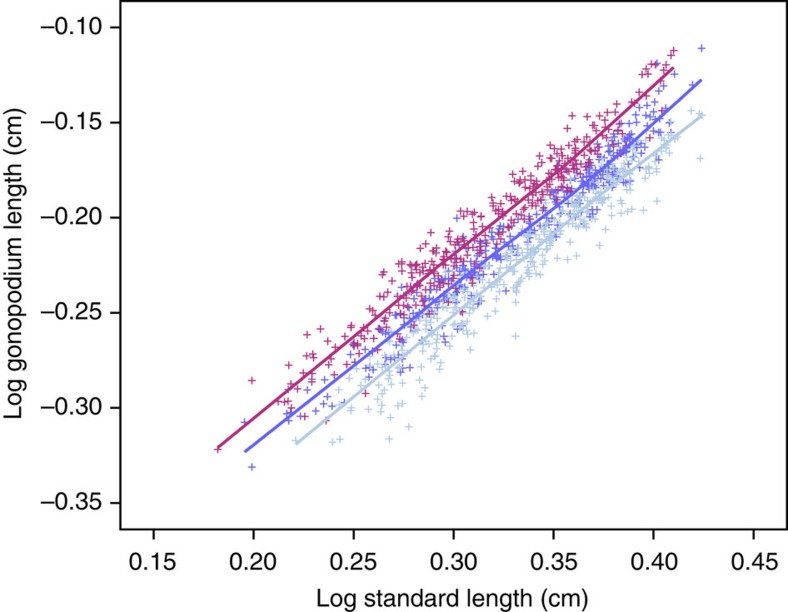
The allometry of gonopodium length for generation 8 males. For clarity, the individual replicates are not shown and the regression lines are based on selection types pooled across replicates (for replicate-specific data, see Supplementary Fig. 4). Purple, Up; dark blue, Control; light blue, Down.

In wild-caught male *G. holbrooki*, body size accounted for 91.2% of variation in gonopodium length (*N*=545). In conjunction with weakly negative allometry (slope of log gonopodium length on log body length regression <1: 0.918±0.012), there is therefore little variation in relative genital size (30.5±0.04% of body length; all summary statistics are mean±s.e.). Despite this precise allometry, we observed a clear response to artificial selection on mean relative gonopodium length (Fig. 1 and Supplementary Fig. 1), indicating no short-term constraints on genital size evolution^37^ (but see refs 22, 23, 31). To test the response of mean relative gonopodium length, male body size and the allometric slope of the gonopodium–body size regression (see Methods for details of response variables) to selection on relative gonopodium length, we ran separate linear models (LMs) for up-selected and down-selected lines for each trait, treating replicate, generation and their interaction as factors. Selection on mean relative genital size resulted in bidirectional evolution (LM: generation: Up: F_1,21_=119.08, *P*<0.001; Down: F_1,21_=67.24, *P*<0.001) that did not differ between replicates (LM: Up: F_2,21_=2.76, *P*=0.09; Down: F_2,21_=1.037, *P*=0.37). Individual regressions of relative gonopodium length on generation were highly significant for all six selection lines (LM: all *P*<0.005, *N*=9 generations, *R*^2^=69.8–89.9%). After eight rounds of selection, the gonopodium of an average-sized male when selected upward was 4.97%, 4.26% and 6.78% larger (replicates *A*, *B* and *C*, respectively), and when selected downward was 3.93%, 3.30% and 2.13% smaller, than that of a control line male (Fig. 2 and Supplementary Fig. 2). This difference persisted after a generation of relaxed selection. The mean relative gonopodium length of up-selected males was 6.66, 3.51 and 4.95% greater, and that of down-selected males was 3.84, 2.95 and 2.73% smaller, than that of control males. The realized heritability of mean relative gonopodium length was 0.028±0.006 in the up-selected lines and 0.022±0.005 in the down-selected lines (Supplementary Table 1). Higher realized heritability estimates are obtained using a less conservative approach (see Supplementary Methods).

The evolution of mean relative gonopodium length was not associated with a change in body size in the down-selected lines (LM: generation: F_1,21_=0.346, *P*=0.563; replicate: F_2,21_=2.10, *P*=0.812), although there was a marginal decrease in body size in the up-selected lines (LM: generation: F_1,21_=4.358, *P*=0.049; replicate: F_2,21_=1.133, *P*=0.341; Supplementary Fig. 3). The allometric slope of gonopodium length did not change in either the up-selected (LM: generation: F_1,21_=0.016, *P*=0.900) or down-selected lines (LM: generation: F_1,21_=0.217, *P*=0.646) nor did this relationship differ among replicates (LM: Up: F_2,21_=2.28, *P*=0.798; Down: F_1,21_=1.758, *P*=0.197; Supplementary Fig. 3). The reduced major axis regression slope was negatively allometric in up-selected (0.891±0.011), down-selected (0.893±0.016) and control lines (0.893±0.011; all *N*=24). This was also true for least-squares regressions.

### Male attractiveness

Male genitalia can directly influence female mate choice in species with external intromittent organs, including poeciliids^11^,^12^ (but see ref. 38) and humans^39^. Artificial selection did not, however, affect male attractiveness, when measured as association times of wild-caught females (*N*=151) that were presented with three size-matched males (control, down- and up-selected; Supplementary Fig. 4). Females spent 39.9±1.2% of each 20 min choice trial associating with compartments housing males and a linear mixed-effects model (LMM) showed that females preferred males over a fourth empty compartment (Wald's *χ*^2^=73.89, *df*=3, *P*<0.0001; Table 1 and Supplementary Table 2). Females spent on average 157.2±12.8 s with the control, 164.5±12.2 s with the down-selected and 157.1±13.5 s with the up-selected male (respectively, 32.6±2.2, 34.7±2.2 and 32.1±2.2% of total association time). Females did not prefer males from up-selected lines that have a relatively long gonopodium for any given body size.

### Male and female swimming performance

Aside from ensuring sperm transfer, natural selection is rarely thought to act directly on male genital size^2^. However, larger genitalia make molting more difficult for arthropods^40^ and population comparisons suggest that predation risk affects gonopodium size and shape in poeciliids^4^,^12^,^41^,^42^ (but see ref. 8). In poeciliids, larger genitalia reduce male burst-swimming speed during antipredator responses, presumably because of increased hydrodynamic drag^12^. We measured the burst-swimming speed of 461 males (21.97±0.18 mm travelled in a 38-ms trial; *N*=49–53 males per line) and 450 females (15.41±0.20 mm per 38 ms; *N*=50 females per line) when startled by a moving stimulus (Supplementary Fig. 5). The distance travelled by males increased by 0.55±0.08 mm for every 1 mm increase in body length (LMM: Wald's *χ*^2^=53.01, *df*=1, *P*<0.0001). It also increased with water temperature (LMM: Wald's *χ*^2^=64.33, *df*=1, *P*<0.0001, Table 2). Artificial selection did not, however, affect male speed (LMM: Wald's *χ*^2^=2.47, *df*=2, *P*=0.29; Tables 2 and 3). Males from down-selected lines were no faster than either control males or those from up-selected lines. For females, warmer temperature increased swimming speed (LMM: Wald's *χ*^2^=6.71, *df*=1, *P*=0.01) but greater body length did not (LMM: Wald's *χ*^2^=1.22, *df*=1, *P*=0.27). Female swimming performance did not evolve as a correlated response to artificial selection on mean relative gonopodium length (LMM: Wald's *χ*^2^=1.04, *df*=2, *P*=0.59; Tables 2 and 3).

### Male reproductive success

**Figure 3:**
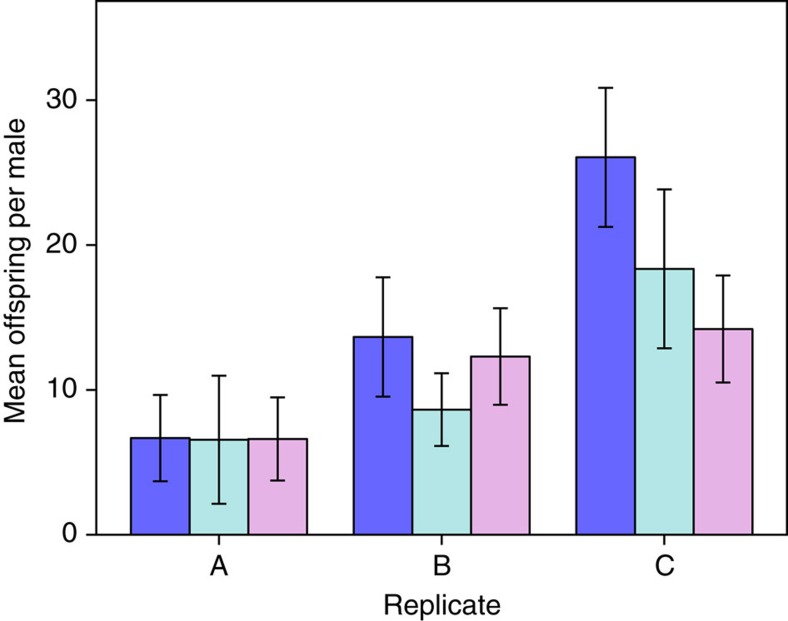
Number of offspring sired per male under semi-natural conditions. Bars indicate±1 s.e.m. Dark blue, Control; light blue, Down; purple, Up. The number of males per selection type per replicate is 18–20. Total *N*=173 males.

To test for sexual selection on relative gonopodium length, we stocked ten large ponds per replicate (700 l; 30 ponds in total) with eight wild-caught virgin females and six males from generation 8. These comprised two trios of size-matched ‘small' and ‘large' males (‘small' were ∼80% the size of ‘large'); each trio consisted of a control, up-selected and down-selected male. We genotyped all males, the 165 females that gave birth and their 2,284 offspring to assign paternity based on ∼4,400 single-nucleotide polymorphisms (see Supplementary Methods). On average, males sired 12.79±1.37 offspring (*N*=173 males). For the 104 males that gained some paternity, the average number of offspring was 26.04±3.11. Neither artificial selection on mean relative gonopodium length nor male size explained how many offspring a male sired (generalized LMM: selection: Wald's *χ*^2^=0.79, *df*=2, *P*=0.68; size: Wald's *χ*^2^=0.11, *df*=1, *P*=0.74; Fig. 3 and Tables 3 and 4). Although mean paternity success appeared to differ across replicates (Fig. 3), replicate was not a significant predictor in the model (generalized LMM: Wald's *χ*^2^=3.27, *df*=2, *P*=0.20; Table 4). If reproductive success was treated as a binary outcome (a male either sired offspring or did not), this *post-hoc* test showed that large males were significantly more likely to sire offspring than were small males (binomial LMM: Wald's *χ*^2^=5.22, *df*=1, *P*=0.02). Again, however, there was no effect of artificial selection on gonopodium length (Table 4). Thus, when males could freely compete for mates and sperm competition occurred, artificial selection revealed no fitness cost associated with the evolution of relatively larger or smaller gonopodia than occur naturally for males of a given body size.

### Body shape

There is strong selection on body shape in fish because of its hydrodynamic effects. For example, population comparisons in *Gambusia* species show convergent evolution of body shape in response to predation risk^43^. We used standard body shape landmarks for fish (Supplementary Fig. 6) in a recently developed geometric morphometric analysis^44^ to test for correlated responses in body shape to artificial selection on gonopodium length (see Supplementary Methods). In both sexes, body shape was related to body length (Procrustes multivariate analysis of variance (MANOVA): males: F_1,660_=66.131, *P*<0.001, *N*=672; females: F_1,418_=19.852, *P*<0.001, *N*=430; Supplementary Fig. 7 and Supplementary Table 3), but this relationship did not change with artificial selection (Procrustes MANOVA: males: F_2,660_=0.964, *P*=0.601; females: F_2,418_=1.819, *P*=0.099; Supplementary Table 3). Controlling for body size, artificial selection on mean relative gonopodium length had a significant correlated effect on body shape (Procrustes MANOVA: males: F_1,660_=11.269, *P*<0.001; females: F_1,418_=10.763, *P*<0.001; Supplementary Table 3). Up-selected, down-selected and control lines all differed significantly from each other for both sexes (all *P*<0.02; Supplementary Table 4). Up-selected males had a deeper body and more posterior gonopodial insertion than down-selected or control line males, and females from up-selected lines had a deeper abdomen and shorter tail than down-selected or control line females (Supplementary Fig. 8). The swimming performance trials indicate, however, that these body shape differences did not affect burst-swimming speed.

### Gonopodium tip shape

Population comparisons in *Gambusia* and other poeciliids reveal relationships between gonopodium tip shape and predation risk^41^, and selection analyses have linked tip shape to male fertilization success^8^. Geometric morphometric analyses showed that tip shape was related to the size of the distal part of the gonopodium (Procrustes MANOVA: F_1,411_=25.44, *P*<0.001; Supplementary Fig. 9 and Supplementary Table 3), which is also highly correlated with total gonopodium length (*r*=0.835, *P*<0.001, *N*=411). Tip shape therefore differed among selection treatments, simply because males differed in gonopodium length (Figs 1 and 2). The allometric relationship between tip size and shape did not differ among selection treatments (Procrustes MANOVA: F_2,399_=1.362, *P*=0.245; Supplementary Table 3). Correcting for size, there were no differences in tip shape in response to artificial selection on gonopodium length (F_2,399_=0.775, *P*=0.647; Supplementary Table 3).

### Fecundity

Natural deviations from the line of allometry might reflect individual variation in quality. For example, many sexual traits are condition dependent, including some genital traits^45^. Greater body condition is likely to have beneficial effects; thus, artificial selection on gonopodium length might have indirectly selected for males in good condition. If condition itself is heritable, we may have indirectly improved mean body condition (of both sexes, if condition has a positive inter-sex genetic correlation). This increase in body condition could elevate female fecundity and/or male fertility. We therefore measured the within-line success of 120 mating pairs per selection treatment. Larger females produced more offspring (generalized LMM: Wald's *χ*^2^=16.14, *df*=1, *P*<0.0001; Table 5 and Supplementary Table 5); however, controlling for replicate (generalized LMM: Wald's *χ*^2^=5.29, *df*=2, *P*=0.07), there was no difference in fecundity between up-selected, control and down-selected pairs (generalized LMM: Wald's *χ*^2^=1.14, *df*=2, *P*=0.57; Table 3). The proportion of pairs that produced offspring varied among selection treatments (control: 83.3%; up-selected: 78.3%; down-selected: 65.8%; each *N*=120); however, after accounting for replicate effects, these differences were not significant (generalized LMM: Wald's *χ*^2^=4.22, *df*=2, *P*=0.12; Supplementary Table 5).

## Discussion

Natural and sexual selection have been proposed to explain key aspects of allometric scaling of male genitals in many taxa, mainly related to allometric slopes^22^,^23^,^46^. Here we focus on the role of selection in explaining another aspect of genital allometry. In wild populations of *G. holbrooki*, there is a very precise relationship between male genital size and body size (*R*^2^>90%). Consequently, there is low variation in relative genital size (that is, genital size corrected for body size). Despite the logistic challenge posed to ensure measurement error does not obscure estimates of a male's relative gonopodium length, we successfully used artificial selection in both directions to produce males with gonopodia that were beyond the naturally observed range of lengths for their body size (Fig. 2 and Supplementary Fig. 2). Therefore, there are no immediate developmental or genetic constraints preventing relative genital size from evolving. Although our estimates of realized heritability are low, we suggest they be treated cautiously. Given stochastic variation in body size and allometric slopes across generations (Supplementary Fig. 2; see also refs 22, 46), there is probably similar variation across lines within generations that introduces noise into estimates of selection differentials and responses to selection, and hence heritability (*h*^2^=*R/S*_c_; see Methods). In contrast, the steady generational change in mean genital size in selected lines (relative to control lines) for an average-sized male is readily apparent (Fig. 1). This steady change is also interpretable in terms of realized heritability, as selection intensity was similar each generation, because we always bred from males in the top/bottom 40 of 129±3 measured males. We applied artificial selection on residuals perpendicular to the allometric line so that genital and/or body size could evolve. As in other studies taking this approach^32^,^33^, the mean value of the focal trait evolved (that is, gonopodium length) while mean body size did not. This difference in response suggests stronger stabilizing selection and/or lower heritability of absolute body size than absolute genital size. As in most studies, there was also an asymmetric response to selection^47^. The response was stronger in up-selected than in down-selected lines (Fig. 1; see also refs 35, 36).

We expect a tight trait–body size relationship with low variation around the line of allometry if there is strong selection for specific trait–body size combinations. For gonopodia, there is evidence from previous studies that selection on relative size could arise from the combined effects of natural and sexual selection. There are several lines of correlational evidence for sexual (or natural) selection on male genitalia. First, comparative analyses show that interspecific genital shape diversity is lower in insect clades where females are monogamous rather than polyandrous, implicating sexual selection^48^. Similarly, the intensity of post copulatory sexual selection sometimes predicts genital evolution^49^. Second, selection analyses show that natural variation in genital size and/or shape predicts paternity in some species^50^,^51^,^52^,^53^,^54^, including *G. holbrooki*^6^,^7^, implying that these traits are sexually selected. Selection analyses in poeciliids also suggest that there is natural selection on genital size, because it affects locomotion, which should affect survival under predation^5^,^20^. Third, experimental evolution studies where sexual selection is present or absent^55^ report that male genitalia evolve as predicted by selection analyses^56^,^57^.

We currently lack experimental studies in which novel genital–body size combinations are created in such a way that developmentally integrated correlated traits can co-evolve. Here we achieved this goal using artificial selection and then tested for selection against deviation from natural allometry. We investigated components of selection acting both directly on genital size and on correlated traits (for example, effects on females arising from inter-sexual genetic correlations). Our most important finding was clear. In a competitive situation where swimming performance, rates of copulation attempts, insemination success and fertilization ability are all likely to affect male reproductive success, there was no detectable effect of deviation from the line of natural allometry (Fig. 3). This key finding is consistent with our detailed investigation of specific potential sources of variation in male fitness. First, despite previous work reporting that a relatively longer gonopodium slows a male's escape response in *Gambusia* species^12^, there was no detectable decline in burst-swimming speed in up-selected males. Second, there was no detectable sexually selected cost of a shorter gonopodium due to reduced male attractiveness. It should be noted that previous experimental evidence for this relationship in *G. holbrooki* is based on cutting 15–17% off the gonopodium^11^, whereas the difference between up-selected and down-selected lines was <9%. Third, there was no change in size-corrected genital tip shape, a trait that predicts fertilization success in other poeciliids^8^.

It is important to note that our main finding of no effect of relative genital size on male reproductive success is unlikely to be due to low statistical power. We assigned 2,284 offspring to 173 potential sires. Three recent selection analyses of *G. holbrooki* and *P. reticulata*, which reported that relative gonopodium length explains significant variation in male reproductive success, were all smaller than our study (Head *et al.*^6^ assigned 844 offspring to 240 potential sires, Vega-Trejo *et al.*^7^ assigned 629 offspring to 122 potential sires and Devigli *et al.*^9^ assigned 532 offspring to 60 males). These studies highlight the discrepancy between our results and past selection analyses. One plausible explanation is that variation in relative gonopodium length in past selection analyses is due to environmental factors that affect fertilization ability. For example, the diet of male *G. holbrooki* affects relative gonopodium size^58^ and sperm production^59^. A similar confounding variable affecting body condition could explain the correlation between swimming performance and relative gonopodium size^12^. It would be useful to test whether the recently reported strong effect of relative gonopodium length on paternity in guppies^9^ is still detected after artificial selection.

Tight allometry of a focal trait need not be due to direct selection on the trait. It might arise due to selection on genetically and developmentally correlated traits (for a possible example, see ref. 35). For example, genital appendages and horn size are genetically correlated in beetles^60^ and optimal horn size depends on body size for biomechanical reasons. Tight genital–body size relationships could similarly arise due to size-dependent allocation of resources to developmentally linked traits under size-dependent selection. For example, ablation of imaginal discs precursory to genitalia increased horn size in dung beetles^61^; thus, environmental variation in resources could yield a genital allometry that is driven by strong size-dependent selection on horns. Selection on other traits should, however, still lead to lower fitness of males selected away from the line of allometry, which we did not observe. Another possibility is that inter-sexual genetic correlations constrain trait allometry. Indeed, in *G. holbrooki*, female body shape did show a correlated response to selection on male genitals but, again, there was no detectable decline in female swimming performance or pair fecundity in selected lines. Finally, we must acknowledge that, as in most studies, we cannot measure net fitness. Instead, we can only measure some components of fitness. It is therefore possible that we failed to detect selection on deviation from the line of natural allometry because we did not measure the appropriate fitness component. Crucially, however, we did measure paternity.

In sum, high variation among species in mean relative genital size is a common pattern in many animal taxa; hence, it is surprising that no previous studies have used artificial selection to alter genital size and test for the effects on fitness. This study design is a powerful way to detect fitness effects of deviation from natural trait–body size relationships (for example, artificial selection on relative butterfly wing size produced males with novel large- or small-winged phenotypes with lower mating success than control males^32^,^33^). Artificial selection increases the available phenotypic variation, which should make it easier to detect fitness costs than when conducting selection analyses on standing variation^62^. This is especially relevant for traits with high *R*^2^ and hence low variation in relative size^42^. The lack of evidence for selection against deviations from the natural line of allometry in our study is therefore a genuine conundrum. Unfortunately, difficulties in reporting unexpected findings lead to well-known publication bias that systematically distorts science^63^. As such, it is difficult to assess whether our results are genuinely anomalous or reflect a larger file drawer problem in evolutionary biology.

## Methods

### Ethics

This research was approved by Australian National University Animal Ethics (Permit F.BTZ.90.05, F.BTZ.26.08 and A2011/64) and New South Wales DPI scientific collection permit (P06/0147-1.0).

### Initiation of experimental lines and selection protocol

We collected eastern mosquitofish (*G. holbrooki*) from Western Sydney, Australia, in March–May 2007. Fish were housed communally in 120-l tanks at 28 °C on a 14:10 h light:dark cycle and fed *ad libitum* (twice daily) with *Artemia* nauplii and fish flakes. We set up 180 gravid females in individual 3-l tanks until we had 150 broods of laboratory-born offspring (there is multiple paternity so the number of sires is >180). To obtain virgin females we continually removed males, who can be identified as soon as their anal fin begins to elongate into a gonopodium. We used 540 virgin females to create 9 experimental lines (60 females per line), with all females within a line originating from a different brood. Given multiple mating in *G. holbrooki*, broods almost always consist of maternal half-siblings. The adult males that we used to initiate lines were field collected in December 2007.

We set up three replicates (*A*, *B* and *C*) between December 2007 and May 2008. Each replicate comprised two selection lines (‘Up' and ‘Down') and an unselected control line. To select founding sires (generation 1) for each replicate, we measured the body size (standard length, SL) and gonopodium length of 121 (*A*), 140 (*B*) and 171 (*C*) wild-caught males. Each male was briefly immobilized in iced water (<4 °C), then photographed with a Nikon Coolpix 5700 digital camera attached to a Leica Wild MZ8 dissecting microscope. Male SL and gonopodium length (from the tip to the juncture between the two last clear segments of the gonopodium before it attaches to the body wall; Supplementary Fig. 6) were measured using ImageJ software (http://imagej.nih.gov/ij/). The allometric relationship for each line was calculated as the reduced major axis (RMA) regression of log gonopodium length on log SL; gonopodium allometry did not differ for the three sets of wild-caught males that initiated the replicates (slopes: F_2,539_=0.469, *P*=0.626; intercepts: F_2,539_=0.247, *P*=0.781). We selected males based on their deviation from the regression line (using positive residuals for up-selected and negative residuals for down-selected males). Selection based on these residuals, which are perpendicular to the regression line, should shift the intercept (that is, mean relative gonopodium size), but not the slope, of the allometry^32^,^33^. This protocol could lead to the evolution of mean relative gonopodium size due to selection on body size and gonopodium size. In contrast, the use of ordinary least squares regression does not generate direct selection on body size. In practice, the use of residuals from ordinary least squares regressions identified almost exactly the same males for selection in every line in every generation. We selected males with the largest (Up) or smallest (Down) relative gonopodium length: 30 males per selection line for replicates *A* and *B*, and 40 males per selection line for replicate *C*. As not all pairs bred, the number of selected males was increased to 40 in all replicates in all subsequent generations to increase the likelihood that at least 30 males successfully sired offspring. For the control lines, 30 (or 40) males were chosen at random from another group of wild-caught males (that is, we did not exclude males that might otherwise have been assigned to an Up or Down line). The least squares regression of log gonopodium length (cm) on log SL (cm) for the 545 wild-caught males was *Y*=0.918±0.012*X−0.491±0.004 (*R*^2^=91.2%). The slope was significantly less than unity (*t*=6.83, *P*<0.001), showing negative allometry. The same was true using RMA regression, where the mean slope was 0.968±0.014 (*R*^2^=90.8%).

Each male was paired consecutively with two virgin females to increase the likelihood that all males sired offspring. Each pair was placed in a 3-l tank for 1 week. The male was then removed, while the female was allowed to produce one to two clutches. Fry were removed from their mothers' tanks on the day of birth; we kept five to ten fry per mother (to obtain ∼10 fry per male) to establish generation 2. Fry were reared in 3-l tanks for ∼1 week (to minimize the risk of early mortality) and then pooled and reared at densities of one to two fish per litre in 120-l tanks. Siblings were split across tanks to minimize any decline in genetic diversity if tanks were lost due to accident. Again, fry were separated by sex at the first signs of maturity, to ensure females remained virgins.

For generations 2–8, once males were mature they were isolated in 1-litre tanks. We measured 129.2±3.1 Up line, 128.5±3.1 Down line and 96.0±3.3 control line males for each generation and replicate (*N*=21 selection events; 7 generations of 3 replicates). For each line, the 40 males with the most positive (Up lines) or most negative (Down lines) residuals, or 40 randomly chosen males from the control line, were selected to sire the next generation (that is, the top or bottom 31% in selected lines, given a mean of 129 measured males). Each selected male was paired consecutively with two randomly chosen females from his line. After reproducing, males were killed and preserved in Dietrich's solution.

The median number of sires per generation was 30 (range: 27–31). Breeding success varied across generations but we kept the number of males contributing to the next generation as similar as possible across lines within each replicate, while ensuring we had ∼300 fry per line. The mean number of actual sires in generations 2–8 when artificial selection was applied was 31.4±0.6 (*N*=63 breeding events; Up: 31.6±0.9, Down: 31.0±1.2, Control: 31.7±1.0, each *N*=21). In generation 9 we did not select males based on relative gonopodium length but randomly used 60 males per line as sires (one female per male), recording their SL and gonopodium length to obtain the population means for this generation. The data for male SL and gonopodium length for generations 1–9 are provided in Supplementary Data 1. Finally, we recorded the SL and gonopodium length of 69.8±5.5 males per line in generation 10, to test whether the observed differences persisted in the absence of selection in the preceding generation (Supplementary Data 2).

### Response to selection

We ran separate general LMs for up-selected and down-selected lines treating replicate, generation and their interaction as factors. There were stochastic environmental effects that affected absolute body size each generation (Supplementary Figs 1 and 3). We therefore decided not to use absolute values of response traits. Instead, following common practice^32^,^33^, we used the deviation of each selection line mean from the control line mean for the relevant generation and replicate. The three response traits were mean gonopodium length, mean body length and the allometric slope (RMA regression) (Supplementary Figs 1 and 3). For gonopodium length, we calculate the value of an average SL (22.18 mm) male from the RMA regression for the relevant generation, replicate and selection line.

We calculated the realized heritability of relative gonopodium length separately for up-selected and down-selected lines, following the methods in ref. 47. The focal ‘trait' was each male's residual from the control allometric slope (RMA regression) in the same replicate, in the same generation. This approach was necessary because of stochastic environmental variation across generations (Supplementary Fig. 3). We regressed the cumulative effective selection differential *S*_c_ against the total response to selection, *R* (the mean of the difference between the expected value of the trait based on the control line in that generation and the observed value for all males in the focal line) (Supplementary Table 1). All six *R* on *S*_c_ regression lines were significantly >0 for up lines and significantly <0 for down lines (all *P*<0.049). Realized heritability (*h*^2^) is twice the value of the regression slope, as we only selected on males. Realized heritability estimates were small (0.016–0.038). We estimated the s.e. of the realized heritability based on the three *h*^2^ estimates per selection regime (up or down), as the use of s.e. associated with each regression line (or pooling the lines) underestimates variation^47^. We calculated the regression using all nine generations because of the approximately linear generational response to selection (Fig. 1) in conjunction with a selection protocol that was very similar across generations and no obvious change in the scatter of residuals around the line of allometry (*R*^2^=90.8±0.7%, *N*=72). The use of all nine data points produced a conservative estimate of *h*^2^. Visual inspection of *R* and *S*_c_ suggested some nonlinearities in the response to selection; excluding later generations improved linearity and increased *h*^2^ estimates (see Supplementary Methods).

### Terminal trait measurement assays

After seven rounds of artificial selection, in 2012 we used individuals from generation 8 to measure the effects of selection on mean relative gonopodium length on morphological, physiological and reproductive (fitness related) traits in both sexes. Assays were performed within replicate, as temporal separation of the *A*, *B* and *C* replicates prevented their combination. The selection lines showed clear divergence in their allometric intercept, but not slope, in all three replicates (Supplementary Fig. 2). We deliberately restricted our analyses to a limited set of traits based on *a priori* justifications that we made at the start of the experiment about the probable effects of a change in relative gonopodium length on these traits. Line means for the assayed traits are presented in Table 3.

### Male attractiveness

We tested whether female preferences differed for males from the three selection regimes. Within each replicate, we created size-matched trios (<0.1 mm SL) with a male from each of the Up, Down and Control lines (*A*: *N*=51 trios, *B*: *N*=58 trios and *C*: *N*=42 trios). The males were individually placed in triangular corner compartments (9 × 9 × 13 cm) of a square choice arena (36 × 36 × 15 cm; Supplementary Fig. 4). The 13-cm wall facing the arena was made of clear Perspex. The fourth corner compartment was empty, to test whether females preferred to associate with males. We randomized male corner positions with respect to selection treatment. The external arena walls were lined with black plastic and the base covered with gravel. Each size-matched trio was used in a single trial. Males were then returned to their tanks. Test females were wild caught as juveniles and maintained in single-sex tanks, to ensure virginity. All females used were sexually mature and previously unmated; hence, they were likely to be receptive to mating. In each trial, a female was placed in a clear plastic cylinder in the centre of the choice arena and allowed to acclimate for 10 min. The cylinder was then raised remotely and her activity recorded for 10 min using a digital video camera positioned directly above the arena. The female was then caught, replaced in the cylinder for 2 min, re-released and recorded for another 10-min period. We used the two 10-min halves of the trials to test the temporal consistency of the female response.

Video analysis was performed blind to the position of each male type. Female preferences were inferred from their association time with each male (see ref. 11), measured as the total time spent <4 cm from his compartment (‘association zone'). We tested for differences in the time females spent in each association zone (that is, with males from each selection treatment or the empty corner) with a linear mixed model in the R package *lme4* (v1.1–9)^64^ with male selection treatment and replicate as fixed effects and trial (female) identity as a random effect (each female provided four data points, one per male and for the empty corner). Again, we did not calculate a random effect for replicate, as it only had three levels. This analysis provides information on the relative time spent with each type of male (Table 1). Females spent only 45.7±5.6 s in the equivalent ‘association zone' of the empty compartment. Rerunning the model excluding the empty corner and including line identity produced almost identical parameter estimates (analysis not shown).

Females spent on average 39.9±1.2% of the 20-min trial associating with males. This ‘total male association time' was not related to the female's size (F_1,146_=0.102, *P*=0.750) or the size of the males available to her (F_1,146_=1.010, *P*=0.317; Supplementary Table 3). Females spent less time associating with males in the second half of the trial (paired *t*-test: *t*_150_=2.68, *P*=0.008). There was, however, still a significant intra-class correlation for time spent with males, indicating that females varied significantly in their propensity to associate with males (*r*=0.27, *N*=151, *P*=0.0003). In addition, individual females showed consistency in the proportion of time they spent with specific males. The intra-class correlations (ICC) for the proportion of the total association time spent with the Control male, the Down male or the Up male, respectively, between the two halves of the trial were all significant (ICC=0.17–0.35, all *N*=151, *P*<0.021). We present analyses for the full 20 min of the trial. The data for female association times are provided in Supplementary Data 3.

### Swimming performance

We tested whether burst-swimming performance (acceleration during a startle response) differed among the three experimental lines. We used 49–53 males and 50 females from each of the 9 lines. Each fish was placed in an opaque plastic tank (24 × 29 cm), with water to a depth of 10–15 mm to limit movement on the vertical plane. A rigid plastic cylinder with a rubber base was suspended in one corner of the tank so that its base just broke the water surface (Supplementary Fig. 5). This stimulus was released when the focal fish was <10 cm from it. It hit the base of the tank, startling the fish so that it performed a ‘C-start' escape response. This response has a characteristic form in which the fish first contracts its lateral musculature to form a C-bend shape^65^. We recorded three consecutive C-starts per fish (*N*=2,733 trials). To calculate the repeatability of burst-swimming behaviour, we re-tested ten males and ten females per line the next day^66^.

Each trial was filmed from above using a digital camera with high-speed video (240 frames per second; Casio Exilim EX-FH100). We analysed the footage frame-by-frame in ImageJ using the plugin MtrackJ (http://www.imagescience.org/meijering/software/mtrackj/) to determine the distance travelled, velocity and acceleration over the first ten frames (38 ms) of the response. The starting point of each track was the position of the fish in the frame that immediately preceded the ‘C'-bend. We recorded the distance from the starting point to the stimulus and the orientation of the fish relative to the stimulus in the starting frame. Preliminary inspection of the data showed that neither factor predicted swimming performance; thus, we excluded them from the final analyses. These video analyses were not performed blind, but the repeatability of our tracking measurements (that is, measurement error) was estimated by re-analysing a random subset of 20 videos.

Although measurement error was small (ICC=0.98, lower, upper 95% confidence interval (95% CI)=0.94, 0.99; F_19,20_=83, *P*<0.0001, *N*=20 videos), the repeatability of retested individuals' behaviour across the six trials was low for both sexes (male: ICC=0.13, 95% CI=0.06, 0.24, F_66,335_=1.9, *P*<0.0001, *N*=67; females: ICC=0.16, 95% CI=0.08, 0.26, F_68,345_=2.1, *P*<0.0001, *N*=69). Thus, to ensure we used the most consistent and meaningful estimate of burst speed performance, for analysis we selected the fastest trial for each fish (the trial with the greatest distance travelled over ten frames)^12^. As the total distance travelled, maximum velocity (greatest distance between consecutive frames) and maximum acceleration (greatest increase in velocity over consecutive frames) were strongly correlated (*r*=0.38–0.94) we restricted our analyses to distance travelled. Owing to the strong sexual size dimorphism in *G. holbrooki* and the need to control for body size, we analysed males and females separately. We used linear mixed models in *lme4* to test whether artificial selection affected burst speed performance. SL, water temperature and replicate were treated as fixed effects, with line identity (nine levels) included as a random effect (Table 2). The data for male and female swimming performance are provided in Supplementary Data 4.

### Male reproductive success

We tested whether selection on gonopodium length affected male paternity success in a semi-natural competitive setting. We set up ten 700-l (1.5 m diameter, ∼40 cm deep) plastic fishponds per replicate housed in a glasshouse at 28 °C. Each pond contained eight adult virgin females (wild-caught as juveniles) and six males: two Up, two Down and two Control. Males were size-matched (<0.1 mm SL) in trios with one male from each line. Each pond contained a trio of large males and a trio of small males (the average size difference between large and small males in a pond was 4.7±1.4 mm; males in the small trio were 80.1±5.2% the size of males in the large trio). We gave each male a unique colour tag with a subcutaneous elastomer implant, injected behind the dorsal fin, while fish were immobilized in iced water (<4 °C).

Fish were left in the ponds to interact freely for 7 (*A*) or 14 (*B* and *C*) days. Males were then photographed for morphometrics (see below) and preserved for genotyping. Females were placed in individual 3-l tanks to produce one to two broods. All fry were individually preserved for genotyping. After ∼10 weeks, mothers were preserved for genotyping.

To assign paternity we genotyped single-nucleotide polymorphism for every female that produced offspring (*N*=165: *A*=32, *B*=64, *C*=69), all potential sires (*N*=179 in total; 1 male from *B* died during the mating period and we could not extract DNA) and every offspring produced from our mating experiments (*N*=2,284 offspring: *A*=369 from 42 clutches, *B*=692 from 89 clutches, *C*=1,223 from 102 clutches). We used the commercial genotyping services of Diversity Arrays Technology who have developed a widely used technique called DArTseq^67^(see Supplementary Methods). We could unambiguously assign paternities for all fry in 29 of the 30 pools. In one pool (replicate *A*) only six fry were produced. They did not match any of the putative sires. The most probable explanation is that the mother had mated and stored sperm before entering the pool. These data were discarded (final *N*=173 potential sires available for analyses).

Owing to high over-dispersion and the fact that only 104 of 173 males gained paternity, we used the number of offspring fathered by a male as the response variable in a zero-inflated negative binomial mixed-effects model, implemented in the R package *glmmADMB* (v0.8.0)^68^. This allows for the inclusion of random effects, but is limited as the zero-inflated part of the model has a constant estimation. However, based on Akaike Information Criterion (AIC) scores it provided a better fit than using negative binomial error without zero inflation (1089.2 versus 1109.6). We included replicate, selection treatment (Up, Down and Control), male size class (small and large) and the treatment × size interaction as fixed effects, and pond identity (29 levels) and line identity (9 levels) as random effects. We included replicate as a fixed rather than random effect, because it only has three levels. In addition, line identity should already account for most of the relevant variation (Table 4). In a second analysis, we treated paternity success as a binary variable (that is, did a male sire any offspring) in a generalized linear mixed model with a binomial error structure and a logit link function, using the same fixed and random effects (Table 4). There was a significant effect of replicate on the likelihood of siring any offspring. Fewer males gained paternity success in replicate *A* compared with *B* or *C* (Wald's *χ*^2^=17.843, *df*=2, *P*=0.0001; Table 4). This is likely to be due to the shorter time allowed for mating in replicate *A*. The data for male reproductive success are provided in Supplementary Data 5.

### Body and gonopodium tip shape

We photographed the left side of anaesthetized fish and digitized ten landmarks per image (Supplementary Fig. 6) for geometric morphometric analysis (see Supplementary Methods). Gonopodia were photographed separately and positioned swung forward to view the distal tip. We measured the length of the entire gonopodium using ImageJ and digitized six landmarks (Supplementary Fig. 6) on the distal portion of the gonopodium, which is the only part inserted into the female. We found vectors that described variation in male body and gonopodium shape, and female body shape using the R package *geomorph* (v2.1.5)^44^. The analysis provides Procrustes coordinates and centroid size for each specimen. To aid visualization and describe variation among fish, we found the axes of major shape variation using principal components analysis of the Procrustes coordinates (Supplementary Figs 7–9). We constructed LMs to determine whether male body shape, female body shape and gonopodium tip shape changed with artificial selection on gonopodium length. In each model the response variable was the two-dimensional set of coordinates that had been aligned using the generalized Procrustes analysis. Selection treatment (Up, Down and Control) and line identity within selection regime were included as factors. We included centroid size as a covariate to control for size-related shape changes (that is, static allometry). Models were run using the *procD.lm* function in *geomorph* (Supplementary Table 3). This performs Procrustes MANOVA with random permutation, to quantify the relative amount of shape variation that is attributable to predictor variables^44^. We then performed *post-hoc* pairwise comparisons of Procrustes distances between least squares means, to determine which selection treatments differed significantly from each other^69^ (Supplementary Table 4). The data and R scripts for male and female body shape are provided in Supplementary Data 6–13 and the data and R scripts for gonopodium tip shape are provided in Supplementary Data 14–17.

### Fecundity

In generation 8, for each of the 9 experimental lines we created 40 within-line pairs and recorded the number of offspring produced in the first brood and, if there was one, a second brood. Females were allowed 7–11 weeks (depending on the replicate) to produce offspring before being killed. We recorded the female SL. We compared the number of offspring produced (in the first brood and in total) using zero-inflated negative binomial mixed-effects models in *glmmADMB*, with replicate, selection treatment and female SL as fixed effects. Line identity was a random effect. We also treated breeding success as a binary variable in a model with binomial error and a logit link, using the same fixed and random effects. The mean number of offspring in the first brood and in total was 7.08±0.53 and 9.28±0.70 for Up, 6.58±0.49 and 10.16±0.76 for Control and 5.42±0.49 and 7.06±0.62 for Down pairs (Tables 3 and 5, and Supplementary Table 5). The data for within-line pair fecundity are provided in Supplementary Data 18.

### Statistics

All statistical tests were conducted using R v3.2.2 (ref. 70) or SPSS 22.0 (IBM Corp., 2013). We used an *α* (significance) level of 0.05 and two-tailed tests. In general, where possible we included line identity as a random factor, because fish from the same selection line are not independent. We include replicate as a fixed effect, as there were too few levels to treat it as a random effect (Bolker *et al.*^71^ recommend at least five to six levels for random effects). Some experimental and artificial selection studies treat only replicate as a random (or fixed) effect and exclude line identity, artificially inflating the sample size. We take a conservative approach and include line identity as a random effect. We present significance tests for fixed effects based on parameter estimates from the full model (that is, *t*=mean/s.e.) and by comparing the full model with one without the term of interest using the *Anova* function in the R package *car*, which provides Wald's *χ*^2^-tests (type III sum of squares) for generalized linear mixed models and F tests for general LMs. Unless otherwise stated, summary statistics and parameters estimated are given as mean±s.e.

### Data availability

Raw data files and R scripts used to generate the reported test statistics and summary statistics are provided as Supplementary Data 1–19. Additional files showing calculations and intermediate steps are available from Dryad (doi:10.5061/dryad.10dk4).

## Additional information

**How to cite this article:** Booksmythe, I. *et al.* Fitness consequences of artificial selection on relative male genital size. *Nat. Commun.* 7:11597 doi: 10.1038/ncomms11597 (2016).

## Supplementary Material

Supplementary Information Supplementary Figures 1-9, Supplementary Tables 1-5, Supplementary Methods and Supplementary References

Supplementary Data Set 1 Male standard length and gonopodium length for all males measured for generations 1 to 9 for all 9 lines.

Supplementary Data Set 2 Male standard length and gonopodium length for all males measured for generation 10 (after one round of relaxed selection) for all 9 lines.

Supplementary Data Set 3 Total female association times with males or an empty 'control' container. Female association times with the Up, Down and Control line males. Data is for all females tested for all 9 lines.

Supplementary Data Set 4 Male and female burst swimming performance for all individuals measured for all 9 lines.

Supplementary Data Set 5 Male reproductive success for all males in all 30 test pools.

Supplementary Data Set 6 File (csv format) to run along with tsp file (Supplementary Data 12) in the provided R script (Supplementary Data 13) to obtain male body shape data for all males measured for all 9 lines.

Supplementary Data Set 7 File (csv format) to run along with tsp file (Supplementary Data 8) in the provided R script (Supplementary Data 9) to obtain female body shape data for all females measured for all 9 lines.

Supplementary Data Set 8 Tsp file to run using R script (Supplementary Data 9) to obtain female body shape data for all females measured for all 9 lines.

Supplementary Data Set 9 R script to run to obtain female body shape data for all females measured for all 9 lines

Supplementary Data Set 10 File (csv format) to run along with tsp file (Supplementary Data 12) in the provided R script (Supplementary Data 13) to obtain male body shape data for all males measured for all 9 lines.

Supplementary Data Set 11 File (csv format) to run along with tsp file (Supplementary Data 12) in the provided R script (Supplementary Data 13) to obtain male body shape data for all males measured for all 9 lines.

Supplementary Data Set 12 Tsp file to run using R script (Supplementary Data 13) to obtain male body shape data for all males measured for all 9 lines.

Supplementary Data Set 13 R script to run to obtain male body shape data for all males measured for all 9 lines

Supplementary Data Set 14 File (csv format) to run along with tsp file (Supplementary Data 16) in the provided R script (Supplementary Data 17) to obtain male gonopodium tip shape for all males measured for all 9 lines.

Supplementary Data Set 15 File (csv format) to run along with tsp file (Supplementary Data 16) in the provided R script (Supplementary Data 17) to obtain male gonopodium tip shape for all males measured for all 9 lines.

Supplementary Data Set 16 Tsp file to run using R script (Supplementary Data 17) to obtain male gonopodium tip shape for all males measured for all 9 lines.

Supplementary Data Set 17 R script to run to obtain male gonopodium tip shape for all males measured for all 9 lines.

Supplementary Data Set 18 The data for fecundity of pairs of fish from the same line for all pairs measured in all 9 lines.

Supplementary Data Set 19 Calculation of realized heritability of residual gonopodium length for Up- and Down-selected lines in replicates A, B and C based on LS regressions.

## Figures and Tables

**Table 1:** Effect of male selection treatment on attractiveness.

**Variable**	**Estimate**±**s.e.**	***t***	**95% CI (lower, upper)**	***χ***^**2**^	***df***	***P*****-value**	**Variance**
Fixed							
Intercept (Control, Rep *A*)	153.86±14.03	10.97	126.36, 181.36				
**Corner**				**73.89**	**3**	**<0.0001**	
Down male	7.26±16.26	0.45	−24.60, 39.12				
Up male	−0.14±16.26	0.01	−32.0, 31.73				
**Empty corner**	**−111.51**±**16.26**	**6.86**	**−143.37**, **−79.65**				
Replicate				2.83	2	0.243	
Replicate *B*	−4.67±13.56	0.35	−31.24, 21.90				
Replicate *C*	18.62±14.72	1.27	−10.22, 47.47				
Random							
Trial							0
Residual							19953

CI, confidence interval.

Attractiveness measured as the time (s) females spent in each ‘association zone' during a 20-min choice trial. Significant effects in bold.

**Table 2:** Effect of selection treatment on burst-swimming performance.

**Variable**	**Estimate**±**s.e.**	***t***	**95% CI (lower, upper)**	***χ***^**2**^	***df***	***P*****-value**	**Variance**
*(a) Males*							
Fixed							
Intercept (Control, Rep *A*)	−9.06±2.88	3.15	−14.71, −3.41				
Selection treatment				2.47	2	0.290	
Down-selected	1.09±0.75	1.47	−0.37, 2.56				
Up-selected	0.91±0.74	1.22	−0.55, 2.37				
**SL (mm)**	**0.55±0.08**	**7.28**	**0.40, 0.70**	**53.01**	**1**	**<0.0001**	
**Water temperature (°C)**	**0.68±0.09**	**8.02**	**0.52, 0.85**	**64.33**	**1**	**<0.0001**	
**Replicate**				**13.85**	**2**	**0.001**	
**Replicate B**	**2.47±0.76**	**3.24**	**0.98, 3.97**				
**Replicate C**	**2.79±0.86**	**3.23**	**1.10, 4.48**				
Random							
Line							0.601
Residual							11.742
							
*(b) Females*							
Fixed							
Intercept (Control, Rep *A*)	4.25±5.04	0.84	−5.63, 14.14				
Selection treatment				1.04	2	0.594	
Down-selected	0.47±1.08	0.44	−1.65, 2.59				
Up-selected	−0.63±1.08	0.58	−2.74, 1.49				
SL (mm)	0.13±0.12	1.11	−0.10, 0.37	1.22	1	0.269	
**Water temperature (°C)**	**0.32±0.12**	**2.59**	**0.08, 0.56**	**6.71**	**1**	**0.01**	
Replicate				4.56	2	0.103	
Replicate *B*	−0.25±1.13	0.22	−2.47, 1.98				
Replicate *C*	−2.13±1.11	1.92	−4.31, 0.05				
Random							
Line							1.42
Residual							15.45

CI, confidence interval; SL, standard length.

Burst-swimming performance measured as the distance travelled (mm) by a fish over the first 38 ms of a startle response (see Methods section ‘Swimming Performance'). Significant effects in bold.

**Table 3:** Line and replicate means for assayed traits.

**Line**	**Paternity: all**		**Paternity: sires only**		**Male attractiveness**		**Male distance**		**Female distance**		**Fecundity**	
	**Mean**±**s.e.**	***N***	**Mean**±**s.e.**	***N***	**Mean**±**s.e.**	***N***	**Mean**±**s.e.**	***N***	**Mean**±**s.e.**	***N***	**Mean**±**s.e.**	***N***
Control A	6.67±2.98	18	15.00±5.54	8	142.08±17.08	51	21.03±0.58	54	17.70±0.50	50	8.78±1.27	40
Down A	6.56±4.42	18	23.60±14.08	5	160.36±21.58	51	21.55±0.57	54	16.13±0.52	50	5.05±1.19	40
Up A	6.61±2.87	18	17.00±5.53	7	140.38±16.83	51	22.50±0.55	53	16.34±0.53	50	7.85±1.25	40
Control B	13.65±4.11	20	21.00±5.33	13	155.36±21.38	58	21.94±0.48	50	15.73±0.55	50	10.55±1.10	40
Down B	8.63±2.51	19	11.71±3.00	14	151.36±18.77	58	23.79±0.55	50	15.53±0.58	50	7.55±1.05	40
Up B	12.30±3.33	20	20.50±4.07	12	141.04±20.59	58	22.00±0.60	50	15.79±0.50	50	12.45±0.95	40
Control C	26.05±4.80	20	30.66±4.83	17	178.27±29.02	42	20.82±0.50	50	13.17±0.62	50	11.15±1.55	40
Down C	18.35±5.49	20	30.58±7.23	12	187.70±24.21	42	22.96±0.46	50	16.04±0.44	50	8.58±0.89	40
Up C	14.20±3.70	20	17.75±4.17	16	199.66±33.27	42	21.16±0.51	50	12.27±0.71	50	7.55±1.29	40
Overall	12.79±1.37	173	26.04±3.11	104	159.62±7.42	453	21.97±0.18	461	15.41±0.20	450	8.83±0.41	360

Paternity is the number of offspring sired by males during competitive mating trials; male attractiveness is measured as female association time (s) in mate choice trials; male and female burst swimming performance are measured as distance (mm) travelled in 38 ms; fecundity is the total number of offspring produced by within-line pairs of generation 8 fish.

**Table 4:** Effect of selection treatment on male reproductive success.

**Variable**	**Estimate**±**s.e.**	***Z***	***P*****-value** ***(Z)***	**95% CI (lower, upper)**	***χ***^**2**^	***df***	***P*****-value**	**Variance**
*a) Number of offspring sired*								
Fixed								
Intercept (Control, Large, Rep *A*)	2.85±0.29	9.89	<0.0001	2.28, 3.41				
Male selection treatment					0.79	2	0.68	
Down-selected	−0.13±0.31	0.42	0.67	−0.74, 0.48				
Up-selected	−0.25±0.31	0.81	0.42	−0.87, 0.36				
Male size category					0.11	1	0.74	
Small	0.11±0.33	0.33	0.74	−0.54, 0.76				
Selection treatment × size category					0.02	2	0.99	
Down × Small	−0.07±0.49	0.13	0.89	−1.02, 0.89				
Up × Small	0.04±0.47	0.08	0.94	−0.89, 0.96				
Replicate					3.27	2	0.20	
Replicate *B*	0.05±0.25	0.16	0.88	−0.52, 0.61				
Replicate *C*	0.47±0.27	1.70	0.09	−0.07, 1.00				
Random								
Pond								2.074 × 10^−9^
Line								2.433 × 10^−9^
								
*b) Paternity gained (yes: 1/no: 0)*								
Fixed								
Intercept (Control, Large, Rep *A*)	0.46±0.52	0.90	0.37	−0.55, 1.47				
Male selection treatment					2.87	2	0.24	
Down-selected	−0.95±0.63	1.51	0.13	−2.19, 0.29				
Up-selected	−0.95±0.63	1.51	0.13	−2.19, 0.29				
**Male size category**					**5.22**	**1**	**0.02**	
**Small**	**−1.43±0.63**	**2.29**	**0.02**	**−2.66, −0.20**				
Selection treatment × Size category					2.25	2	0.33	
Down × Small	0.72±0.85	0.85	0.39	−0.94, 2.38				
Up × Small	1.27±0.85	1.50	0.13	−0.39, 2.93				
**Replicate**					**17.84**	**2**	**0.0001**	
**Replicate B**	**1.26±0.41**	**3.09**	**0.002**	**0.46, 2.06**				
**Replicate C**	**1.72±0.43**	**4.05**	**<0.0001**	**0.89, 2.56**				
Random								
Pond								2.331 × 10^−3^
Line								2.602 × 10^−12^

CI, confidence interval; GLMM, generalized linear mixed-effects model.

Male reproductive success measured as (a) the number of offspring sired; (b) whether the male sired any offspring or not (binary variable: yes=1, no=0). Untransformed parameter estimates from a zero-inflated negative binomial GLMM with log link. Significant effects in bold.

**Table 5:** Predictors of the breeding success of pairs from different selection treatments.

**Variable**	**Estimate±s.e.**	***Z***	***P*****-value** ***(Z)***	**95% CI (lower, upper)**	***χ***^**2**^	***df***	***P*****-value**	**Variance**
Fixed								
Intercept (Control, Rep *A*)	−1.99±1.10	1.81	0.07	−4.16, 0.17				
Selection treatment					1.14	2	0.07	
Down-selected	−0.08±0.09	0.92	0.36	−0.26, 0.09				
Up-selected	0.04±0.09	0.43	0.67	−0.13, 0.21				
**Female SL (mm)**	**0.15±0.04**	**4.02**	**<0.0001**	**0.08, 0.22**	**16.14**	**1**	**<0.0001**	
Replicate					5.29	2	0.07	
**Replicate** ***B***	**0.17±0.09**	**1.89**	**0.06**	**−0.01, 0.35**				
Replicate *C*	0.13±0.09	1.43	0.15	−0.05, 0.31				
Random								
Line								1.653 × 10^−08^

CI, confidence interval; GLMM, generalized linear mixed-effects model; SL, standard length.

Breeding success measured as the total number of fry produced by a pair, that is, first and, if it occurred, second brood (see Supplementary Table S5 for additional measures of breeding success). Untransformed parameter estimates from a zero-inflated negative binomial GLMM with log link. Significant effects in bold.
