# Supplementary material for: Fitness consequences of artificial selection on relative male genital size
Source: Nat Commun. 2016 May 18;7:11597. doi: 10.1038/ncomms11597 (PMC4873965; doi:10.1038/ncomms11597)
Supplement: Supplementary Information — Supplementary Figures 1-9, Supplementary Tables 1-5, Supplementary Methods and Supplementary References [file ncomms11597-s1.pdf]

Supplementary Figures

a

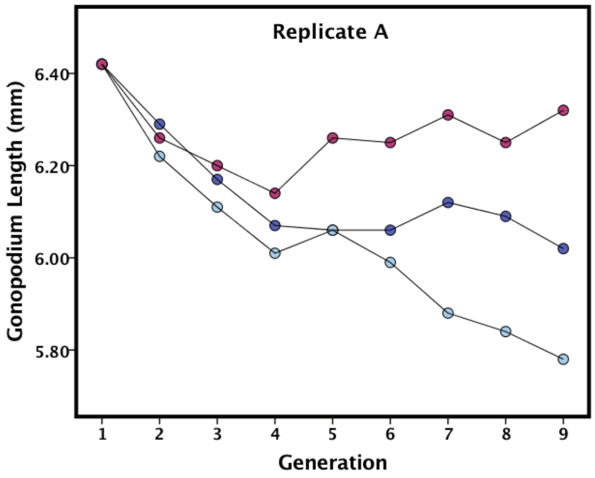

b

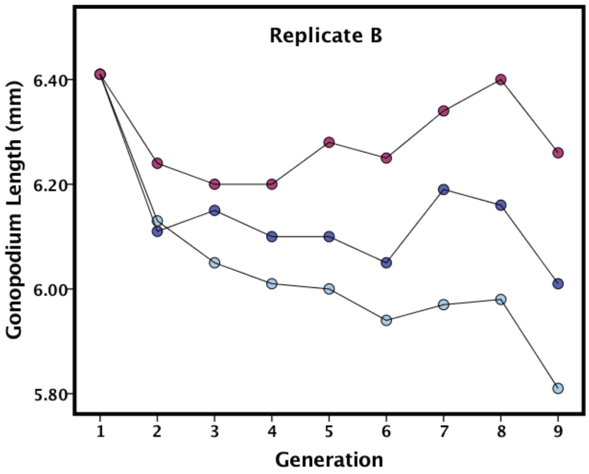

c

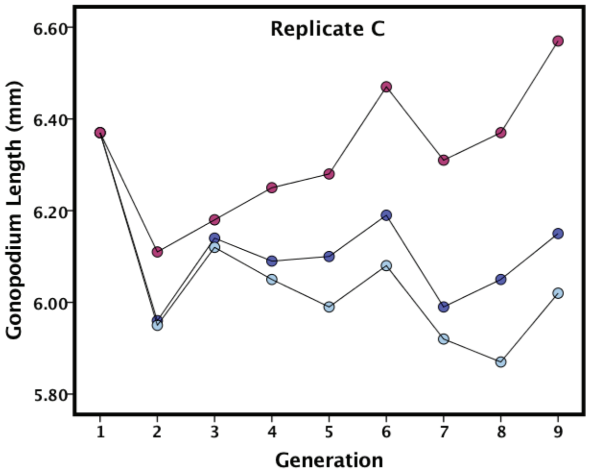

Supplementary Figure 1. Response to selection on relative gonopodium length. Absolute values for a male of mean size (22.18mm) using least squares means from a general linear model of generations 1-9, with each unique generation-replicate treated as a factor (75 levels); a) Replicate A, b) Replicate B, c) Replicate C. Purple = Up, Dark blue = Control, Light blue = Down. The line mean estimates are precise due to the high R^2 values and large sample sizes per generation so, for clarity, SE bars are not presented (they are all < 0.02mm). N = 151-215 males per replicate in generation 1; N = 72-147 males per generation for lines in generations 2-8; N = 54-60 males per line in generation 9. The mean ± SE males for generations 2-8 is 96 ± 3.3 for controls, 128.6 ± 3.1 for Down lines and 129.2 ± 3.1 for Up lines.

a

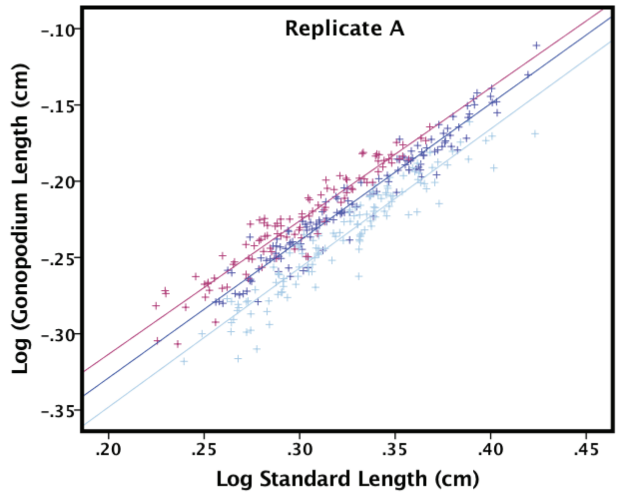

b

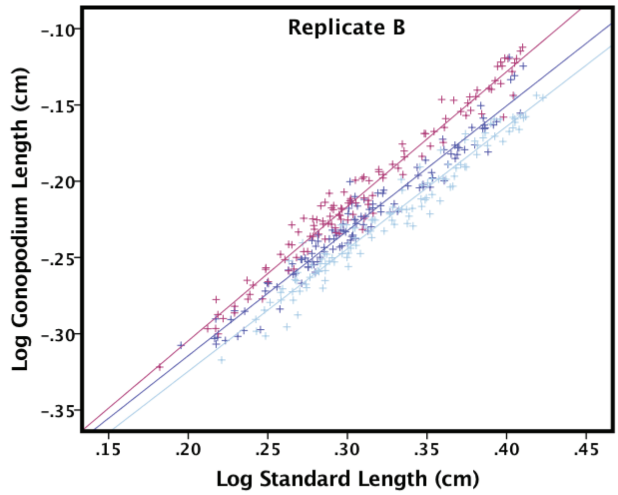

c

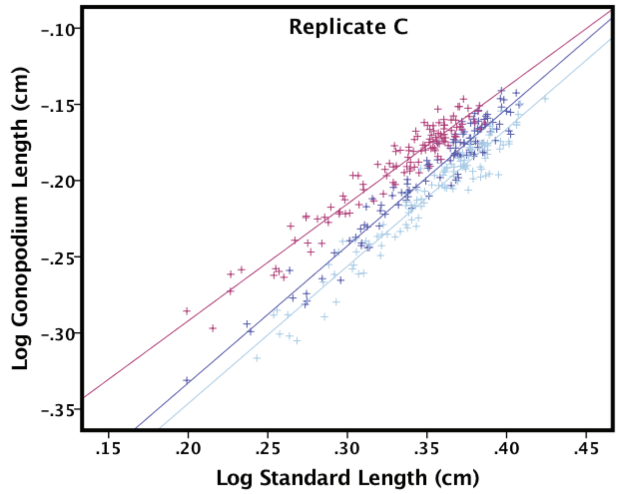

Supplementary Figure 2. The allometry of gonopodium length after seven rounds of selection (generation 8); (**a**) Replicate A (N = 130, 130, 101), (**b**) Replicate B (N = 120,119,119), (**c**) Replicate C (N = 119,120,117) (N for Control, Down, Up respectively)**.** There is no difference in the allometric slope between the three selection treatments (GLM: $F_{2,1069} = 1.058$, $p = 0.348$). Purple = Up; Dark blue = Control; Light blue = Down.

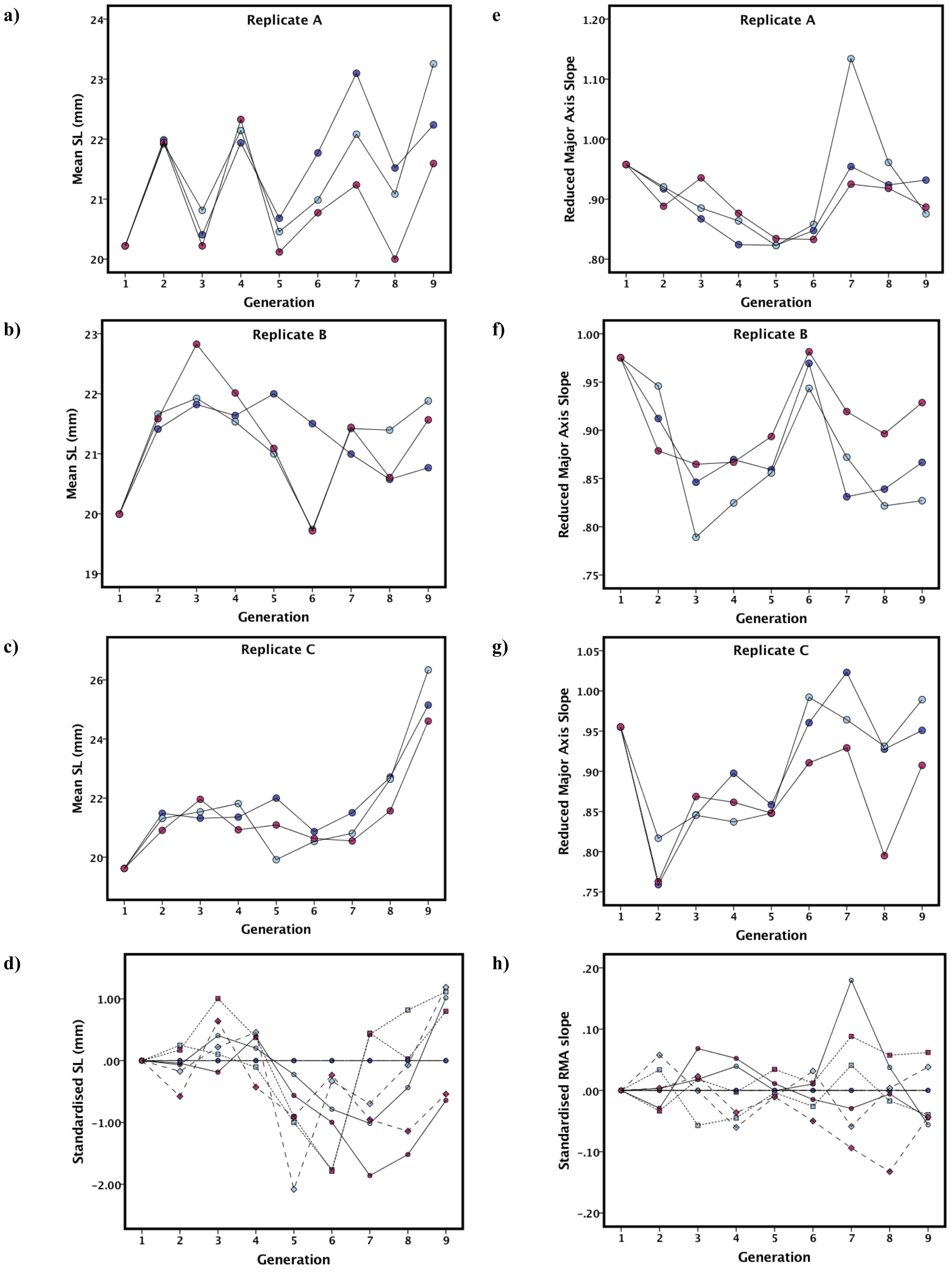

Supplementary Figure 3. Correlated response of male standard length (SL) and gonopodium allometry to artificial selection. **(a-c)** Absolute values for SL for Replicates A-C, respectively; **(d)** Deviations (in mm) from the mean control SL each generation, Rep A: circles and solid line, Rep B: squares and dotted line; Rep C: diamonds and dashed line; **(e-g)** Absolute values (slope coefficients for a reduced major axis regression of log gonopodium length on log SL) for gonopodium allometry for Replicates A-C, respectively; **(h)** Deviations from control slope each generation (symbols as in **d**). Purple = Up, Dark blue = Control, Light blue = Down. (Sample sizes in Supplementary Fig 1).

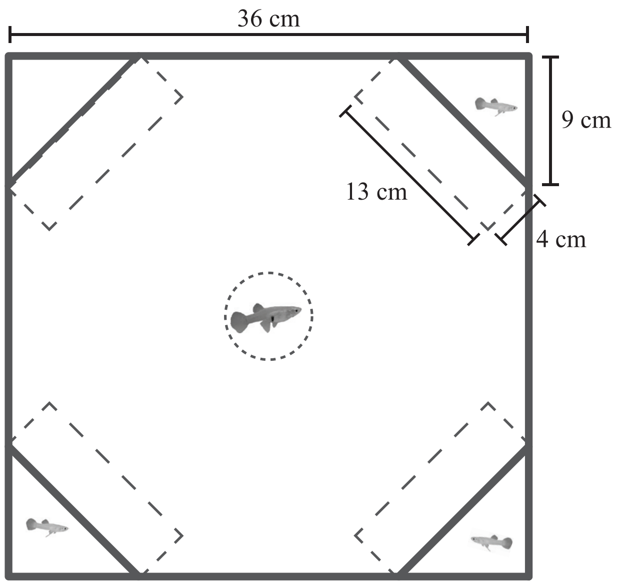

Supplementary Figure 4. Mate choice tank design. Solid lines depict Perspex tank walls and internal barriers. Dashed lines indicate ‘association zones’ of the corner compartments. Females were only considered to associate with males while within one of these zones. Dotted line shows the position of the clear plastic tube in which the female was acclimated, which was removed to release the female at the start of the trial.

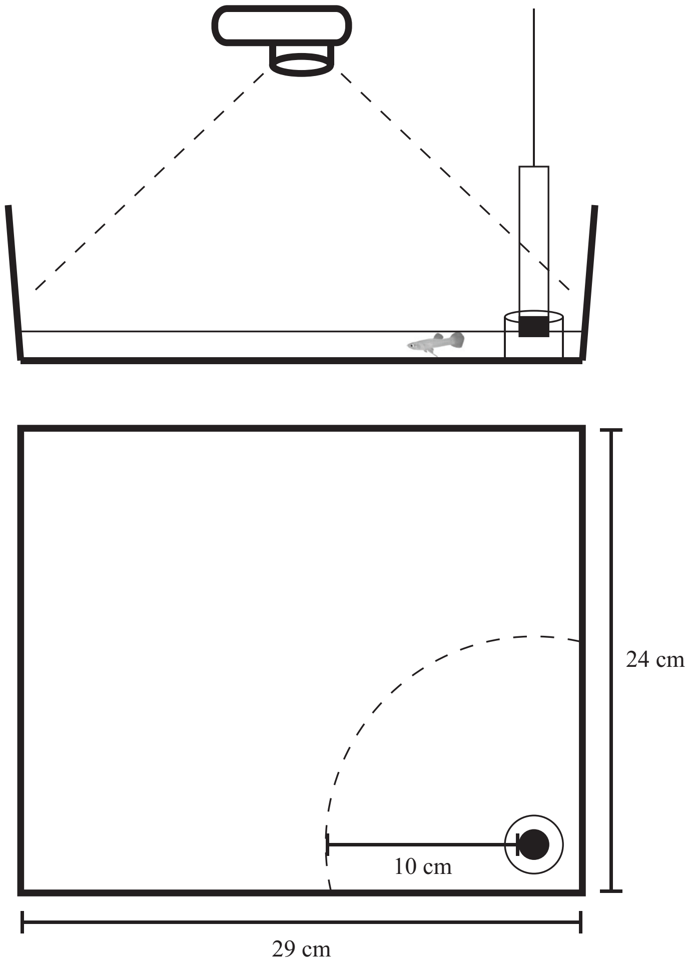

Supplementary Figure 5. Burst-swimming performance tank design. Top: side view. The camera was positioned and zoom level chosen (the same settings in every trial) so that the base of the tank filled the viewfinder and the fish could be tracked anywhere in the tank. Water depth was 10-15 mm (thin horizontal line). Bottom: birds-eye view. The 'startle' stimulus (black circle) was released only when the test fish was within 10 cm (dashed arc). The stimulus was dropped within a clear plastic barrier to protect the fish from contact with it; the startling effect was due to the visual perception of movement and the impact of the rubber base on the floor of the tank.

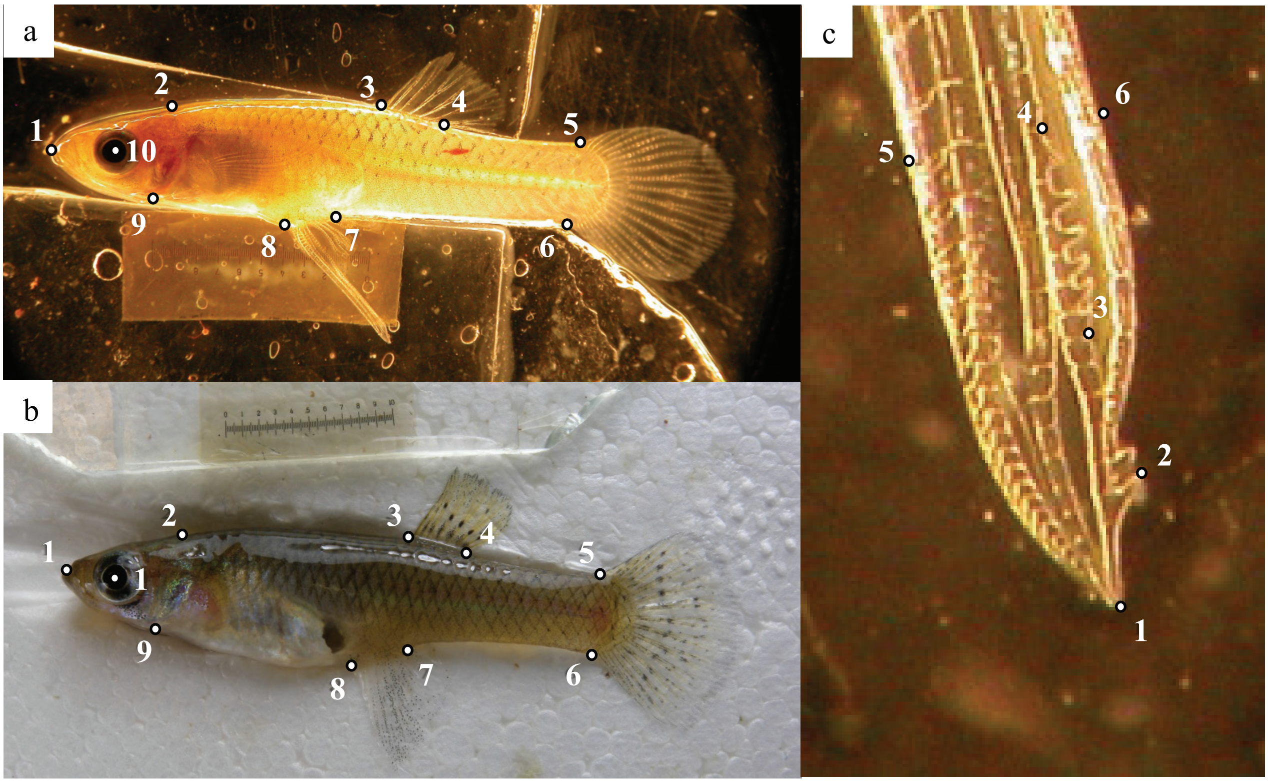

Supplementary Figure 6. Landmarks for morphological analysis of: (**a**) male body shape; (**b**) Female body shape; (**c**) distal tip of the gonopodium. For male and female body shape we digitized the following landmarks: 1) the most anterodorsal point of the premaxilla (tip of snout); 2) the indentation at the posterodorsal end of the head; 3) the anterior insertion of the dorsal fin; 4) the posterior insertion of the dorsal fin; 5) the dorsal insertion of the caudal fin; 6) the ventral insertion of the caudal fin; 7) the posterior insertion of the anal fin; 8) the anterior insertion of the anal fin; 9) the intersection of the operculum and the ventral midline of the body; 10) the centre of eye. For male genital morphology we digitized the following landmarks: 1) the distal tip of the gonopodia; 2) the tip of the distal hook; 3) the distal edge of the midline hooks; 4) the proximal edge of the midline hooks; 5) the anterior edge of the gonopodium at the level of landmark four, perpendicular to the midline; 6) the posterior edge of the gonopodium at the level of landmark four, perpendicular to the midline. The 1cm scale bar, divided into 1 mm then 0.1 mm sections is visible in (**a**) and (**b**).

(a)

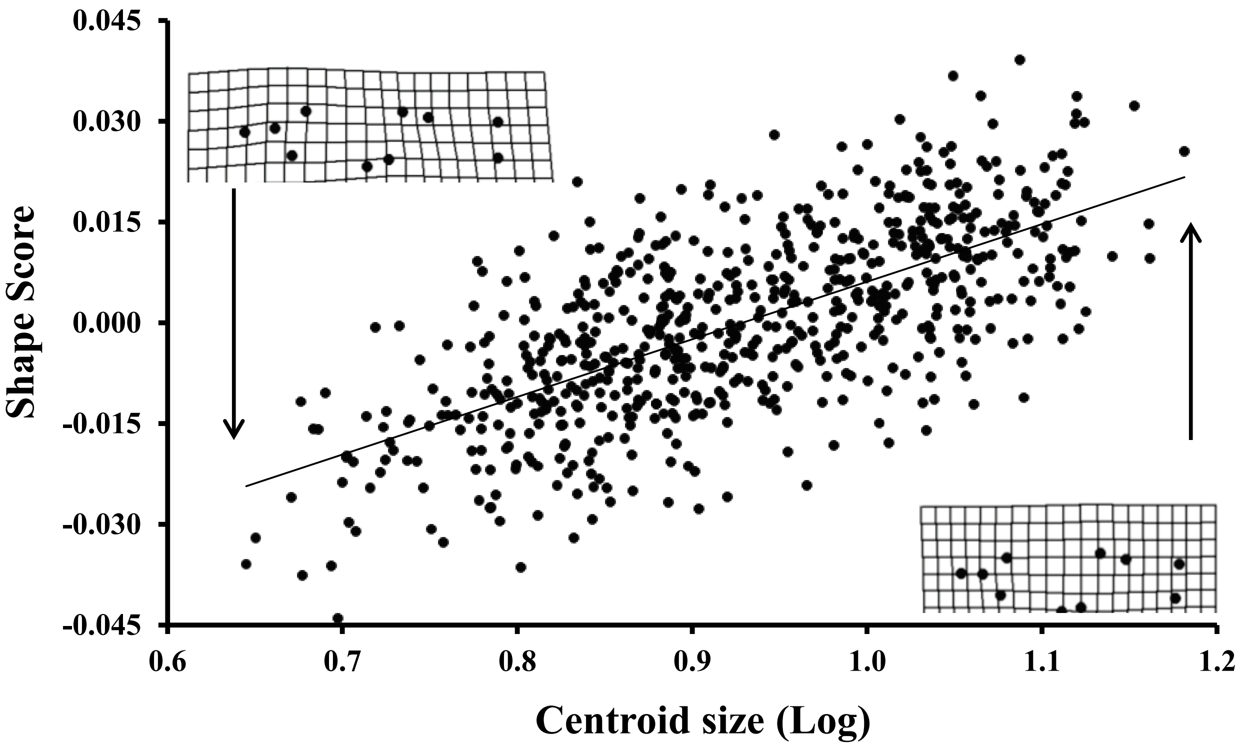

(b)

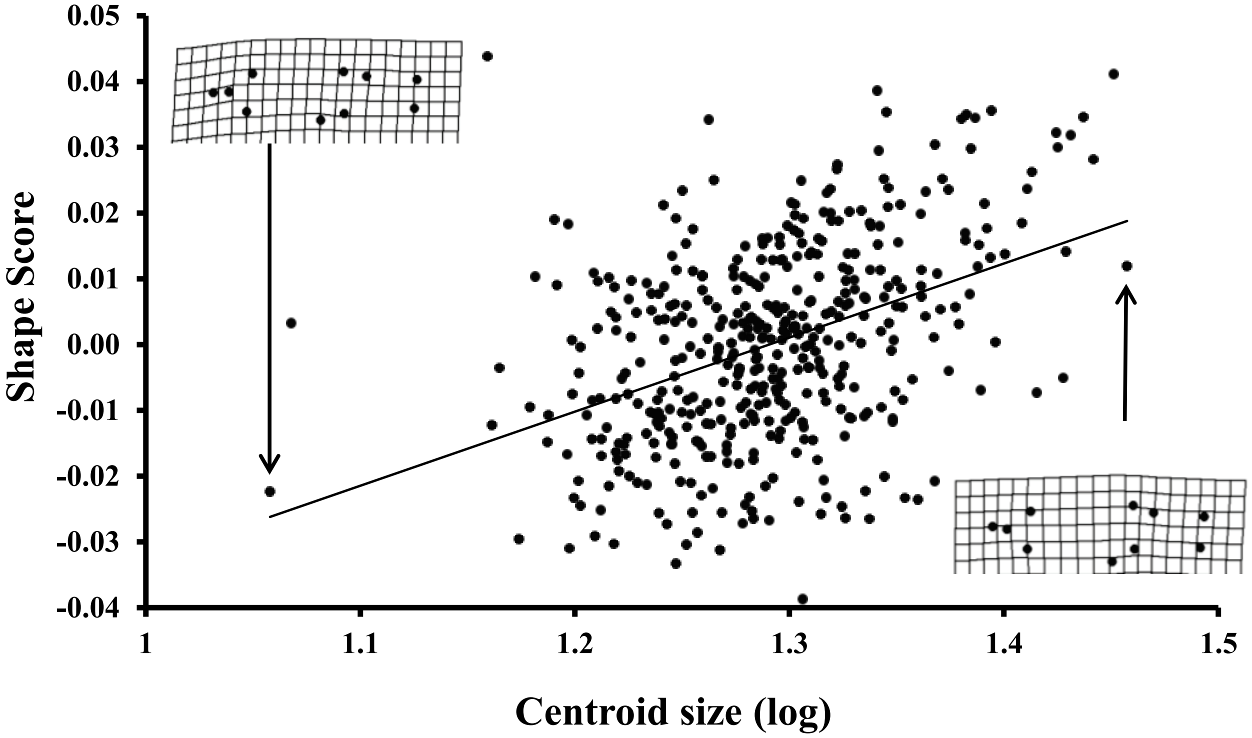

Supplementary Figure 7. The relationship between body size and shape. (a) Males and **(b)** females. Thin plate splines show deviation from the overall consensus shape.

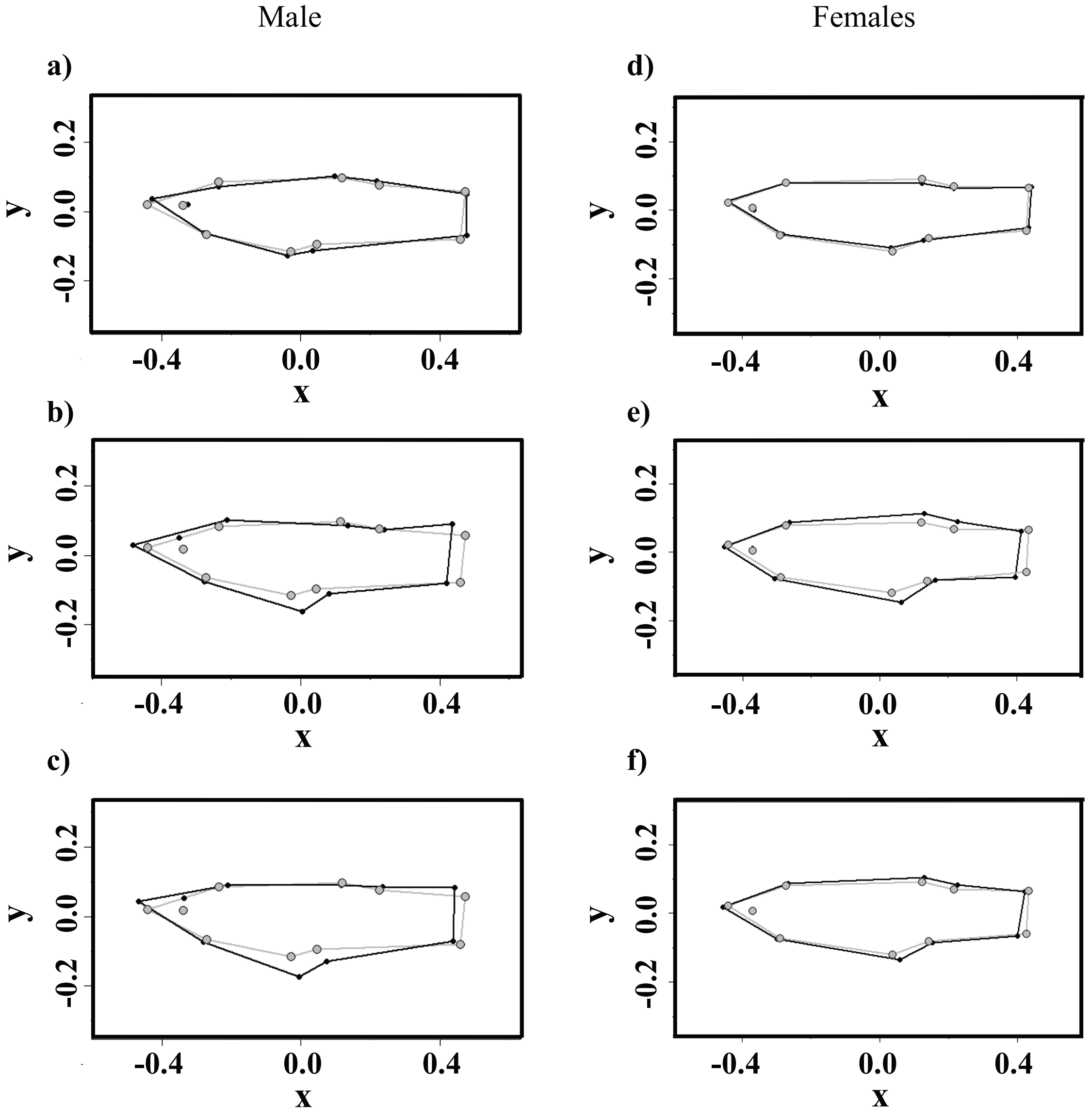

Supplementary Figure 8. Mean body shape for each selection treatments. Male shape differences are magnified by 10x to aid visualization. The shape of: (**a**) Control (grey) and Down (black) males; (**b**) Down (grey) and Up (black) males; (**c**) Control (grey) and Up (black) males. Female shape differences are magnified by 5x to aid visualization. The shape of (**d**) Control (grey) and Down (black) females; (**e**) Down (grey) and Up (black) females; (**f**) Control (grey) and Up (black) females. x and y axes represent standardized shape co-ordinates.

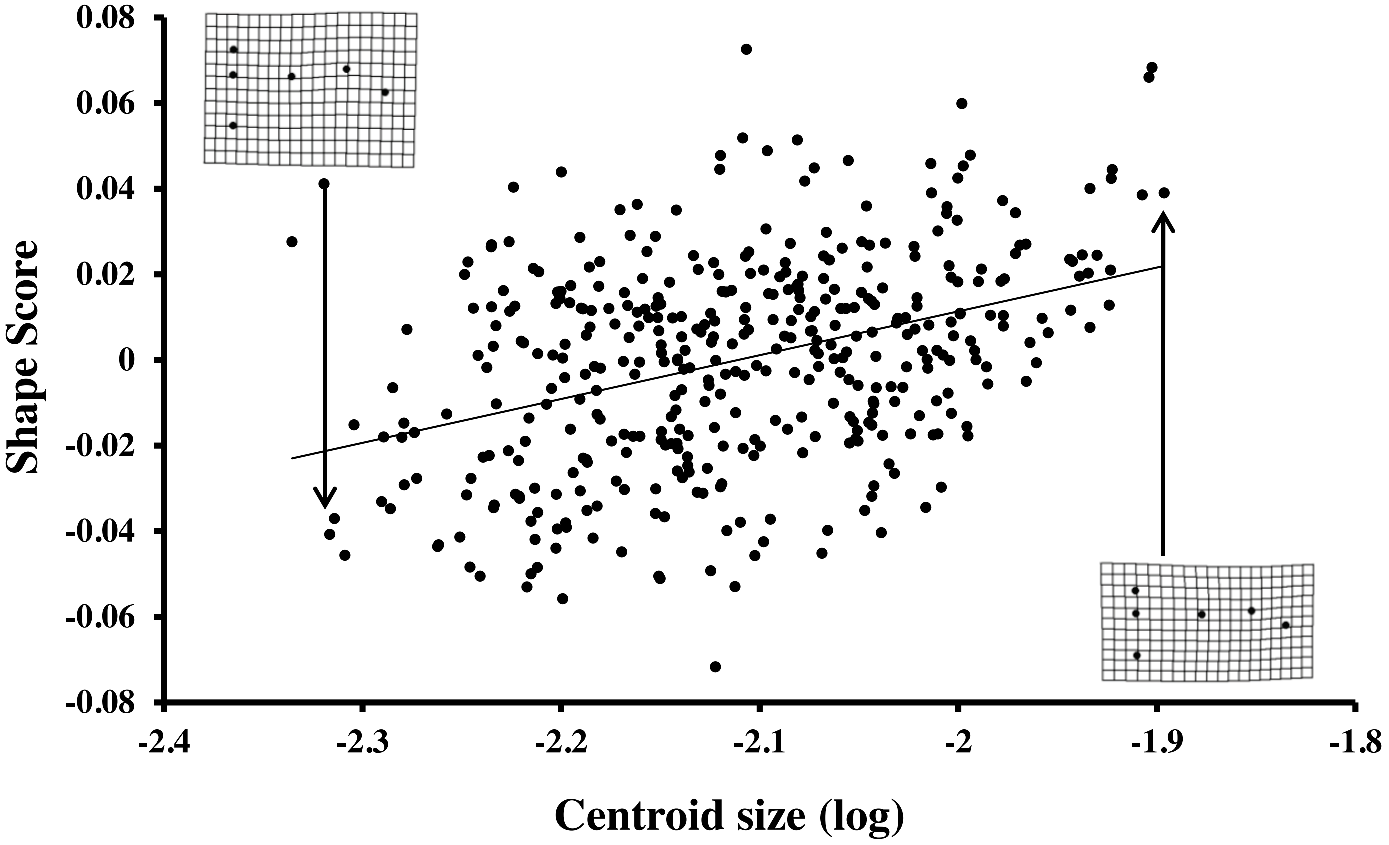

Supplementary Figure 9. The relationship between gonopodium tip size and shape. Thin plate splines show deviation from the overall consensus shape.

Supplementary Tables

Supplementary Table 1. Calculation of realized heritability of residual gonopodium length for Up- and Down-selected lines in replicates A, B and C.

	Up-selected lines			Down-selected lines		
**Generation**	***R***	$S_{\text{cumulative}}$	h^2	***R***	$S_{\text{cumulative}}$	h^2
**Rep A**						
1	0	0		0	0	
2	-1.59591E-05	0.000212534		-5.09849E-05	-0.000182793	
3	2.02247E-05	0.000270505		-4.90054E-05	-0.000289077	
4	3.87603E-05	0.000574944		-4.01826E-05	-0.000535176	
5	0.000147068	0.001160785		1.07017E-05	-0.001092279	
6	0.000155352	0.002381709		-6.40705E-05	-0.002198324	
7	0.000211115	0.004830391		-0.000257563	-0.00445168	
8	0.000149416	0.009665439		-0.000229206	-0.00885885	
9	0.000310661	0.019229491	**0.028**	-0.000271941	-0.017771754	**0.032**
**Rep B**						
1	0	0		0	0	
2	0.000115156	0.000146798		5.27476E-06	-0.000136295	
3	2.49859E-05	0.000288		-5.85372E-05	-0.000240637	
4	7.55245E-05	0.000513261		-5.88222E-05	-0.000494436	
5	0.000137907	0.001077417		-5.28952E-05	-0.001012495	
6	0.000225707	0.002166492		-2.95571E-05	-0.001978854	
7	0.000115337	0.004446617		-0.000173565	-0.00396074	
8	0.00021028	0.008792663		-0.000133903	-0.008013671	
9	0.000225093	0.017634681	**0.019**	-0.000159748	-0.01600177	**0.019**
**Rep C**						
1	0	0		0	0	
2	0.000193355	9.63032E-05		-9.43398E-06	-0.000105396	
3	2.10855E-05	0.000439177		-1.19879E-05	-0.000275278	
4	0.000116525	0.000617856		-1.32552E-05	-0.000447112	
5	0.000233899	0.00131133		-8.08221E-05	-0.000896386	
6	0.00030432	0.002628944		-8.80258E-05	-0.001857416	
7	0.000368473	0.005463103		-4.48413E-05	-0.003802577	
8	0.000367069	0.010920318		-0.000138784	-0.007514821	
9	0.000509227	0.021864925	**0.038**	-0.000124618	-0.01503416	**0.016**

The focal trait was the male's residual deviation from the control-line allometric slope in the same replicate and generation. *R*: response to selection (mean trait value of generation *t* males), $S_{\text{cumulative}}$: cumulative selection differential (sum from generation 1 to *t*-1 of the difference between mean trait value of all males and weighted mean of selected males), h^2: realized heritability for each line and replicate (= 2*a* where $R = a \cdot S_{\text{cumulative}} + b$). Mean ± SE h^2 Up = 0.028 ± 0.006, Down = 0.022 ± 0.005. These values are based on RMA regressions. Additional values based on LS regressions are given in.Supplementary Data 19.

Supplementary Table 2. **Predicting the total time (s) females spent with males during a choice trial.**

	Estimate ± SE	*t*	95% CI (lower, upper)	*F*	*df*	*p*
Intercept	162.52 ± 398.33	0.408	-624.72, 949.76			
Female standard length (mm)	-2.97 ± 9.29	0.320	-21.33, 15.39	0.102	1,146	0.75
Mean male standard length (mm)	17.92 ± 17.83	1.005	-17.32, 53.16	1.010	1,146	0.32
Replicate				6.019	2,146	0.003
Replicate B	334.70 ± 329.04	1.017	-315.60, 984.99			
Replicate C	447.20 ± 324.84	1.377	-194.80, 1089.20			

Supplementary Table 3. MANOVAs of male and female body shape and gonopodium tip shape.

	df	SS	MS	*F*	*p*
**a) Male body shape**					
**Log centroid size**	**1**	**0.062**	**0.062**	**66.131**	**0.001**
**Selection treatment**	**2**	**0.021**	**0.011**	**11.269**	**0.001**
**Selection treatment/Line**	**3**	**0.026**	**0.009**	**9.379**	**0.001**
Selection treatment*Log centroid size	2	0.002	0.001	0.964	0.601
Selection treatment/Line*Log centroid size	3	0.005	0.002	1.844	0.078
Residuals	660	0.619	0.001		
Total	671	0.736			
**b) Female body shape**					
**Log centroid size**	**1**	**0.017**	**0.017**	**19.852**	**0.001**
**Selection treatment**	**2**	**0.018**	**0.009**	**10.763**	**0.001**
**Selection treatment/Line**	**3**	**0.035**	**0.012**	**14.209**	**0.001**
Selection treatment*Log centroid size	2	0.003	0.002	1.819	0.099
Selection treatment/Line*Log centroid size	3	0.003	0.001	1.394	0.249
Residuals	418	0.348	0.001		
Total	429	0.424			
**c) Gonopodium tip shape**					
**Log centroid size**	**1**	**0.036**	**0.036**	**25.444**	**0.001**
Selection treatment	2	0.002	0.001	0.775	0.647
Selection treatment/Line	3	0.008	0.003	1.979	0.060
Selection treatment*Log centroid size	2	0.004	0.002	1.362	0.245
Selection treatment/Line*Log centroid size	3	0.007	0.002	1.626	0.135
Residuals	399	0.560	0.001		
Total	410	0.617			

P values are from permutation tests (see Supplementary methods section "Body and Gonopodium Tip Shape"). Significant effects in bold.

Supplementary Table 4. Pairwise Procrustes distances between least squares means for male and female body shape.

**Males**	Control	Down	Up	**Females**	Control	Down	Up
Control		**0.018**	**0.001**	Control		**0.019**	**0.001**
Down	0.005		**0.001**	Down	0.006		**0.001**
Up	0.012	0.013		Up	0.010	0.015	

The absolute differences between selection treatments are below the diagonal. The associated *P* values are above the diagonal (based on randomized residual permutation procedure with 1000 random permutations). Significant *P* values in bold.

Supplementary Table 5. Predictors of the breeding success of pairs from different selection treatments.

**Variable**	**Estimate ± SE**	***Z***	***p (Z)***	**95% CI (lower, upper)**	X^2	***df***	***p***	**Variance**
**(a) First brood**								
Fixed								
Intercept (Control, Rep A)	-2.02 ± 1.16	1.74	0.08	-4.29, 0.25				
Selection treatment					5.01	2	0.08	
Down selected	0.09 ± 0.10	0.95	0.34	-0.10, 0.28				
**Up selected**	**0.19 ± 0.09**	**2.12**	**0.03**	**0.01, 0.37**				
**Female standard length (mm)**	**0.13 ± 0.04**	**3.52**	**0.0004**	**0.06, 0.21**	**12.41**	**1**	**0.0004**	
Replicate					**1.50**	**2**	**0.47**	
Replicate B	0.11 ± 0.09	1.22	0.22	-0.07, 0.30				
Replicate C	-0.001 ± 0.10	0.01	0.99	-0.19, 0.19				
Random								
Line								5.806×10^{-09}
**(b) Breeding success (yes: 1 / no: 0)**								
Fixed								
Intercept (Control, Rep A)	-3.84 ± 3.89	0.99	0.32	-11.46, 3.78				
Selection treatment					4.22	2	0.12	
Down selected	-0.95 ± 0.51	1.88	0.06	-1.94, 0.04				
Up selected	-0.15 ± 0.53	0.28	0.78	-1.18, 0.89				
Female standard length (mm)	0.16 ± 0.13	1.28	0.20	-0.09, 0.41	1.65	1	0.12	
**Replicate**					**6.09**	**2**	**0.048**	
**Replicate B**	**1.30 ± 0.53**	**2.47**	**0.01**	**0.27, 2.32**				
Replicate C	0.59 ± 0.49	1.19	0.23	-0.38, 1.55				
Random								
Line								0.222

Breeding success measured as **(a)** the number of fry in the first brood only; **(b)** the production of any offspring or not (binary variable: yes = 1, no = 0). Significant effects in bold.

Supplementary Methods

Heritability of relative gonopodium length

We calculated the realized heritability of relative gonopodium length separately for up-selected and down-selected lines[1]. The focal 'trait' was each male's residual from the control-line allometric slope (RMA regression) in the same replicate in the same generation. This approach was necessary because of stochastic environmental variation across generations (Supplementary Fig. 3). When calculating the selection differential, S (i.e. the difference in residual scores between selected males and all measured males in that generation from that line) we weighted each selected male by his proportional contribution to the offspring that were pooled to establish the next generation (i.e. the effective selection differential)[1]. In one case (Replicate A, generation 6) we had to re-pair fish after females had been returned to communal tanks so we could not directly link selected males to how many offspring they sired. We therefore assume that each selected male contributed the same number of offspring. This is equivalent to using the *expected* selection differential[1]. In the other 71 cases we calculated the effective selection differential.

We regressed the cumulative selection differential $S_{\text{cumulative}}$ against the total response to selection, R (the mean of the difference between the expected value of the trait based on the control line in that generation and the observed value for all males in the focal line)[1]. All six R on $S_{\text{cumulative}}$ regression lines were significantly greater than zero for up-selected lines and significantly less than zero for down-selected lines (all $P < 0.049$). Heritability (h^2) is twice the value of the regression slope as we only selected on males. Heritability estimates were small (0.016-0.038; mean ± SE: Up = 0.028 ± 0.006, Down = 0.022 ± 0.005; Supplementary Table 1). We estimated the standard error of the heritability based on the three h^2 estimates per selection type, because use of the SE associated with each regression line (or pooling the lines) underestimates the SE[1].

We calculated the regression using all nine generations because of the approximately linear generational response to selection (Fig. 1) in conjunction with a selection protocol that was very similar across generations and no obvious change in the scatter of residuals around the line of allometry (R^2= 90.8 ± 0.7%, n = 72). However, visual inspection of R and $S_{\text{cumulative}}$ suggested some non-linearities in the response to selection. The response to selection appeared negligible after generation 6 especially in up-selected lines, and before generation 3 in down-selected lines. Running the regressions using generations 1-6 increased h^2 estimates (Up = 0.175 ± 0.011; Down = 0.049 ± 0.028) while estimates were slightly lower using generations 3-9 (Up = 0.025 ± 0.005; Down = 0.019 ± 0.005).

Paternity assignment (terminal assay: male reproductive success)

To quantify paternity we SNP genotyped every female that produced offspring (N = 165: A = 32, B = 64, C = 69), all potential sires from each pool (6 per pool, 179 in total – one male from B died during the mating period and we could not extract DNA) and every offspring produced from our mating experiments (N = 2284 offspring: A = 369 from 42 clutches, B = 692 from 89 clutches, C = 1223 from 102 clutches). We used the commercial genotyping services of Diversity Arrays (DArT) who have developed a widely used technique called DArTseq™. It represents a combination of complexity reduction methods and next generation sequencing platforms[2-5]. It is an implementation of sequencing complexity-reduced representations[6] on next generation sequencing platforms[7,8]. It is optimized for each species using the most appropriate complexity reduction method based on both the size of the representation and the fraction of the genome assayed. Four methods of complexity reduction were tested for *Gambusia* and the PstI-HpaII method was selected. DNA samples were processed in digestion/ligation reactions principally following Kilian *et al.*[2], but replacing a single PstI-compatible adaptor with two adaptors corresponding to different Restriction Enzyme (RE)

overhangs. The PstI-compatible adapter was designed to include Illumina flowcell attachment sequence, sequencing primer sequence and ‘staggered’, varying length barcode region, similar to the sequence reported by Elshire *et al.*[8]. The reverse adapter contained a flowcell attachment region and HpaII-compatible overhang sequence. Only ‘mixed fragments’ (PstI-HpaII) were effectively amplified in 30 rounds of PCR using the following reaction conditions: 1. 94°C for 1 min; 2. 30 cycles of 94°C for 20 sec, 58°C for 30 sec, 72°C for 45 sec; 3. 72°C for 7 min. After PCR equimolar amounts of amplification products from each sample of a 96-well microtiter plate were bulked and applied to c-Bot (Illumina) bridge PCR, followed by sequencing on an Illumina Hiseq2500. The sequencing (single read) was run for 77 cycles. (EBPCRI primer: 5’- AATGATACGGCGACCAC CGAGATCTACACTCTTTCCCTACACGACGCTCTTCCGATCT - 3’ and EBHpaIIpcr primer: 5’- CAAGCAGAAGACGGCATACGAGATCGGTCTCGGCATTCCTGCTGAACCGCTCTTCC GATCTCGG - 3’).

Sequences generated from each lane were processed using proprietary DArT analytical pipelines. In the primary pipeline the fastq files were processed to filter out poor quality sequences, applying more stringent selection criteria to the barcode region than the rest of the sequence. In that way the assignments of the sequences to specific samples carried in the ‘barcode split’ step are very reliable. Approximately 2,500,000 (± 7%) sequences per barcode/sample are used in marker calling in routine DArTseq assay, but we applied a more cost effective version of the assay using half this number (average of 1.3 million/sample). Finally, identical sequences were collapsed into ‘fastqcall files’. These files were used in the secondary pipeline for DArT PL’s proprietary SNP and SilicoDArT (presence/absence of restriction fragments in representation) calling algorithms (DArTsoft14). This clean up process resulted in a comprehensive data set of approximately 4465 SNPs with an average call rate of 93.5% and a reproducibility rate of 98.8%. From these selected SNPs we calculated a Hamming Distance Matrix for all 2566 individuals to determine paternity. Recent studies show that as few as 30 optimized SNPs are sufficient to differentiate among 100,000 individuals based on Hamming Distance values[9]. All fry were lined up against their mother and siblings and the Hamming Distance values evaluated to cross check for potential sample mix-ups. There were none. Hamming Distance values were then compared against each of the six potential sires and the sire/fry with the lowest value was considered a match. We could unambiguously assign paternities for all fry in 29 of the 30 pools. In one pool only six fry were produced and they did not match any of the putative sires. The most likely explanation is that the mother of these fry had mated and stored sperm prior to being introduced to the pool. The data from this pool (from replicate A) were discarded.

Geometric morphometrics (body and gonopodium tip shape)

We took digital photographs of the left side of anaesthetized fish (females: Nikon Coolpix P6000; males: Nikon Coolpix 5700 attached to a Leica Wild MZ8 dissecting microscope). We digitized 10 landmarks per image (Supplementary Fig. 6) using *tpsDig*[10]. Landmarks were selected for their ease of localization and because they capture key aspects of shape variation in *Gambusia* species[11-13]. Separate photographs of gonopodia were taken. It was swung forward to view the distal tip. We measured the length of the entire gonopodium using *ImageJ* (http://imagej.nih.gov/ij/) and digitized six landmarks (Supplementary Fig. 6) using *tpsDig*. These were confined to the distal portion of the gonopodium because it is the only part inserted into the female[14]. Again, these landmarks have been shown to capture important components of gonopodium shape in Poeciliids[15,16].

We conducted geometric morphometric analysis of male and female body shape, and the morphology of the distal tip of the male gonopodium using the digitized landmarks. In each case we found vectors that described shape variation using the R package *geomorph* (v2.1.5)[17]. This program uses generalized Procrustes analysis to scale each fish (or gonopodium) to a common size (centroid size of 1), center all specimens to the origin and optimally rotate specimens using a least squares criterion for alignment. Consequently, differences in size or positioning do not contribute to

shape differences between specimens[18,19]. The analysis provides Procrustes coordinates and centroid size for each specimen. To aid visualization and describe variation among fish we found the axes of major shape variation using principal components analysis of the Procrustes coordinates (Supplementary Figs. 7-9). These principal components are equivalent to the relative warps produced using other geometric morphometric software such as *TpsRelW*[20].

We constructed linear models to determine whether male body shape, female body shape, and gonopodium tip shape changed with selection on gonopodium length. In each model the response variable was the two-dimensional set of coordinates that had been aligned using the generalized Procrustes analysis. Selection treatment (Up, Down, Control) and line identity within selection regime were included as factors. We included centroid size as a covariate to control for size-related shape changes (i.e. static allometry). Models were run using the *procD.lm* function in *geomorph* (Supplementary Table 3). This performs Procrustes MANOVA with random permutation to quantify the relative amount of shape variation that is attributable to predictor variables[17]. We then performed post-hoc pairwise comparisons of Procrustes distances between least squares means to determine which selection treatments differed significantly from each other[21] (Supplementary Table 4).

Supplementary references

1. Falconer, D.S. & Mackay, T.F.C. *Quantitative Genetics*. Harlow: Pearson Education Ltd. (1996).
2. Kilian, A., Wenzl, P., Huttner, E., Carling, J., Xia, L., Blois H, Caig, V., Heller-Uszynska, K., Jaccoud, D., Hopper, C., Aschenbrenner-Kilian, M., Evers, M., Peng, K., Cayal, C., Hok, P. & Uszynski, G. Diversity array technology: a generic genome profiling technology on open platforms. *Meth. Mol. Biol.* **888**, 67–89 (2012).
3. Courtois, B., Audebert, A., Dardou, A., Roques, S., Ghneim- Herrera, T., Droc, G., Frouin, J., Rouan, L., Gozé, E., Kilian, A., Ahmadi, N. & Dingkuhn, M. Genome-wide association mapping of root traits in a Japonica rice panel. *PLoS One* **8**, e78037 (2013).
4. Cruz, V.M., Kilian, A. & Dierig, D.A. Development of DArT marker platforms and genetic diversity assessment of the U.S. collection of the new oilseed crop lesquerella and related species. *PLoS One* **8**, e64062 (2013).
5. Raman, H., Raman, R., Kilian, A., Detering, F., Carling, J., Coombes, J., Diffey, S., Kadkol, G., Edwards, D., McCully, M., Rupaerao, P., Parkin, I.P., Batley, J., Luckett, D.J. & Wratten, N. Genome-wide delineation of natural variation for pod shatter resistance in *Brassica napus*. *PLoS One* **9**, e101673 (2014).
6. Altshuler, D., Pollara, V.J., Cowles, C.R., Van Etten, W.J., Baldwin, J., Linton, L. & Lander, E.S. An SNP map of the human genome generated by reduced representation shotgun sequencing. *Nature* **407**, 513-516 (2000).
7. Baird, N.A., Etter, P.D., Atwood, T.S., Currey, M.C., Shiver, A.L., Lewis, Z.A., Selker, E.U., Cresko, W.A. & Johnson, E.A. Rapid SNP discovery and genetic mapping using sequenced RAD markers. *PLoS One* **3**, e3376 (2008).
8. Elshire, R.J., Glaubitz, J.C., Sun, Q., Poland, J.A., Kawamoto, K., Buckler, E.S. & Mitchell, S.E. A robust, simple genotyping-by-sequencing (GBS) approach for high diversity species. *PLoS One* **6**, e19379 (2011).
9. Hu, H., Liu, X., Jin, W., Ropers, H.H. & Wienker, T.F. Evaluating information content of SNPs for sample-tagging in re-sequencing projects. *Sci. Rep*. **5**, 10247 (2015).
10. Rohlf, F.J. Tpsdig and tpsrelw programs. Available from http://life.bio.sunnysb.edu/morph (1997).
11. Langerhans, R.B., Layman, C.A., Shokrollahi, A.M. & DeWitt, T.J. Predator-driven phenotypic diversification in *Gambusia affinis*. *Evolution* **58**, 2305-2318 (2004).
12. Langerhans, R.B., Gifford, M.E. & Joseph, E.O. Ecological speciation in *Gambusia* fishes. *Evolution* **61**, 2056-2074 (2007).
13. Langerhans, R.B. & DeWitt, T.J. Shared and unique features of evolutionary diversification. *Am. Nat.* **164**, 335-349 (2004).
14. Rosen, D.E. & Gordon, M. Functional anatomy and evolution of male genitalia in Poeciliid fishes. *Zoologica* **38**, 1-47 (1953).
15. Evans, J.P., Gasparini, C., Holwell, G.I., Ramnarine, I.W., Pitcher, T.E. & Pilastro, A. Intraspecific evidence from guppies for correlated patterns of male and female genital trait diversification. *Proc. R. Soc. B* **278**, 2611-2620 (2011).
16. Evans, J.P., van Lieshout, E. & Gasparini, C. Quantitative genetic insights into the coevolutionary dynamics of male and female genitalia. *Proc. R. Soc. B* **280**, 20130749 (2013).
17. Adams, D.C. & Otarola-Castillo, E. geomorph: an R package for the collection and analysis of geometric morphometric shape data. *Methods Ecol. Evol.* **4**, 393-399 (2013).
18. Gower, J.C. Generalized Procrustes analysis. *Psychometrika* **40**, 33-51 (1975).
19. Rohlf, F.J. & Slice, D.E. Extensions of the Procrustes method for the optimal superimposition of landmarks. *Syst. Zool.* **39**, 40-59 (1990).
20. Rohlf, F.J. Shape statistics: Procrustes superimpositions and tangent spaces. *J. Classification* **16**, 197-223 (1999).
21. Collyer, M.L., Sekora, D.J. & Adams, D.C. A method for analysis of phenotypic change for phenotypes described by high-dimensional data. *Heredity* **115**, 357–365 (2015).
